# Structure of the large terminase from a hyperthermophilic virus reveals a unique mechanism for oligomerization and ATP hydrolysis

**DOI:** 10.1093/nar/gkx947

**Published:** 2017-10-24

**Authors:** Rui-Gang Xu, Huw T. Jenkins, Alfred A. Antson, Sandra J. Greive

**Affiliations:** York Structural Biology Laboratory, Department of Chemistry, University of York, York YO10 5DD, UK

## Abstract

The crystal structure of the large terminase from the *Geobacillus stearothermophilus* bacteriophage D6E shows a unique relative orientation of the N-terminal adenosine triphosphatase (ATPase) and C-terminal nuclease domains. This monomeric ‘initiation’ state with the two domains ‘locked’ together is stabilized via a conserved C-terminal arm, which may interact with the portal protein during motor assembly, as predicted for several bacteriophages. Further work supports the formation of an active oligomeric state: (i) AUC data demonstrate the presence of oligomers; (ii) mutational analysis reveals a *trans*-arginine finger, R158, indispensable for ATP hydrolysis; (iii) the location of this arginine is conserved with the HerA/FtsK ATPase superfamily; (iv) a molecular docking model of the pentamer is compatible with the location of the identified arginine finger. However, this pentameric model is structurally incompatible with the monomeric ‘initiation’ state and is supported by the observed increase in *k*_cat_ of ATP hydrolysis, from 7.8 ± 0.1 min^−1^ to 457.7 ± 9.2 min^−1^ upon removal of the C-terminal nuclease domain. Taken together, these structural, biophysical and biochemical data suggest a model where transition from the ‘initiation’ state into a catalytically competent pentameric state, is accompanied by substantial domain rearrangements, triggered by the removal of the C-terminal arm from the ATPase active site.

## INTRODUCTION

Most dsDNA bacteriophages and viruses utilize a powerful DNA translocation motor to package their unit-length genome into a preformed procapsid (1,2). A central component of the motor, large terminase, consists of an N-terminal adenosine triphosphatase (ATPase) domain comprising ASCE (Additional Strand Catalytic glutamate) and lid subdomains, and a C-terminal nuclease domain (3,4), connected by a short linker sequence. While both domains are involved in interaction with DNA (5,6) DNA translocation is powered by the ASCE subdomain using energy released coinciding with ATP hydrolysis (7,8). Due to topological differences between viral large terminases and other ASCE ATPases, they were classified into a unique division within the superfamily comprised of HerA/FtsK, RecA and PilT ATPases (9,10). The nuclease domain is responsible for cleavage of the genomic DNA concatemer at both the initiation and completion stages of viral DNA packaging (11). This domain is a member of the RNase H-like endonuclease superfamily and has the highest similarity with the RuvC endonucleases (12–14).

Unlike hexameric ATPases (10,15), evidence suggests that the large terminase motor likely assembles into pentameric rings (3,5,16,17). The mechanism of the ATPase motor assembly and how ATP hydrolysis is coupled to DNA translocation, remain poorly understood. Various models have been proposed, including the electrostatically-driven mechanism (3), the chemo-mechanical coupling model (4) and the pentameric *trans-*finger model (5). According to the electrostatic model proposed on the basis of T4 large terminase (3) ATP hydrolysis within ASCE subdomain induces rotation of the lid subdomain (also referred to as ‘transmission domain’) that triggers electrostatic pulling of the nuclease domain towards the ATPase domain, translocating DNA (3). However, according to the chemo-mechanical coupling model based on structural studies of the Sf6 large terminase, DNA is translocated by the conformational changes of the lid (‘linker’) subdomain upon ATP hydrolysis (4). Notably, both these models involve the participation of a *cis*-acting P-loop arginine (*cis-*arginine) to trigger the movement of the lid subdomain upon ATP hydrolysis. Recently, a pentameric *trans*-acting arginine finger (*trans*-arginine) model has been reported for the phi29 packaging ATPase and P74–26 large terminase ATPase. Distinct to the previous two models, this model compares the large terminase ATPase motor to multi-subunit ASCE proteins where a conserved *trans*-arginine finger from the adjacent subunit couples ATP hydrolysis within a pentameric assembly (5). The *trans*-arginine finger (R146) of phi29 was identified at the equivalent position as that reported for FtsK (18). This is consistent with previous comparative genomic studies which classified the phi29 packaging ATPase as a distinct branch within the HerA/FtsK superfamily of the ASCE ATPases (10). Similarly, the P74–26 large terminase also involves a *trans*-arginine residue (R139) that drives ATP hydrolysis from the adjacent subunit (5). This residue was found at the same surface but on a different strand compared to that observed for phi29. In addition, it was also proposed that, for the P74–26 large terminase, the P-loop arginine emphasized by the previous models is analogous to the sensor II of AAA+ proteins and drives the movement of the lid subdomain upon ATP hydrolysis *in cis* (5).

Here, we report the crystal structure of the wild-type (WT) large terminase protein from a thermophilic *Geobacillus stearothermophilus* bacteriophage, D6E, isolated from deep-sea vents (19). In this structure, the N-terminal ATPase and the C-terminal nuclease domains, tethered by a linker, adopt vastly different relative orientations to those observed in large terminases from other bacteriophages. Structures of complexes with bound adenosine diphosphate (ADP) and ATP-γ-S provide the structural basis for ATPase activity. In contrast to P74–26 large terminase, and in common with the phi29 packaging ATPase, the identified *trans*-arginine finger, R158, is located in the same loop as for the HerA/FtsK superfamily. Structural observations, along with striking differences in biochemical activity between the full-length protein, C-terminal mutants and the ATPase domain, indicate that motor assembly must be accompanied by significant conformational rearrangements with the C-terminal arm stabilizing the catalytically inactive but high ATP affinity monomeric ‘initiation’ state.

## MATERIALS AND METHODS

### Cloning, expression and purification

The DNA fragment encoding the full length D6E large terminase (residues 1–427) was synthesized (Genewiz USA Inc.) with codons optimized for *Escherichia coli* protein expression. This DNA fragment was amplified by PCR and re-cloned into the expression vector pET-YSBLIC3C using ligation-independent cloning (20) resulting in a sequence encoding for a protein with an N-terminal 6-histidine tag fused to the human rhinovirus 3C protease cleavage site. Site directed mutagenesis was used to introduce a stop codon for the ATPase domain construct (1–234) or codon changes for mutant variants of the full-length large terminase mutants using the CloneAmp™ HiFi PCR Premix (Takara Bio USA, Inc). The full-length terminase, ATPase domain and all mutants were expressed in *E. coli* BL21-Gold (DE3) (Agilent Technologies USA, Inc) cultured in LB medium containing 30 μg/ml kanamycin. Cells were grown at 37°C until OD_600_ reached 0.6–0.8 followed by induction with 1 mM isopropyl 1-thio-β-D-galactopyranoside (IPTG) and further growth for 2 h before harvesting by centrifugation for 20 min at 5000 × *g* at 4°C. Pellets were frozen at -80°C until purification.

Before sonication, cell pellets of the D6E large terminase proteins were resuspended in buffer A (20 mM Tris pH 7.5, 1 M NaCl) containing 1 mM AEBSF, 0.5 μg/ml leupeptin, 0.7 μg/ml pepstatin and 0.1 mg/ml lysozyme. The lysate was clarified by centrifugation at 19 000 × *g* for 1 h and filtration using a 0.45 μm filter. Proteins were first purified by nickel affinity chromatography with a His-Trap column (GE Healthcare) equilibrated with buffer A containing 10 mM imidazole, and eluted with a 10–500 mM imidazole linear gradient in buffer A. The eluted target protein fractions were collected and dialyzed into 20 mM Tris pH 7.5, 250 mM NaCl, at 4°C overnight. During the dialysis, HRV 3C protease was added to the protein in a 1:100 (w/w) ratio to remove the N-terminal 6-His-tag. Digested protein samples were applied to the His-Trap column as before, the flow-through concentrated and applied to a Superdex 200 Hiload 16/60 column pre-equilibrated in 20 mM Tris–HCl, pH 7.5, and 250 mM NaCl (buffer B). The ATPase domain was purified as the full-length protein, except that 1M NaCl was used in dialysis buffer and buffer B. The final protein samples were concentrated to 10–40 mg/ml, snap frozen in liquid nitrogen and stored at −80° until further use.

### Analytical ultra-centrifugation

Sedimentation velocity experiments were performed to analyse the oligomeric state of the full-length D6E large terminase protein. Purified protein (at a concentration of 4 μM) was dialyzed extensively against a buffer containing 10 mM HEPES pH 7.5 and 50 mM potassium acetate using a 3500 Da cut-off dialysis device prior to the experiment. The dialyzed protein sample together with the dialysis buffer, used as a reference, were pipetted into sector-shaped cells with quartz windows and analysed by a Beckman Optima XL-A analytical ultracentrifuge. The sample was centrifuged at 35 000 rpm at 20°C and radial scans at a wavelength of 280 nm were obtained continuously. Sedimentation profiles were analysed using Sedfit (21) using a partial specific volume of 0.74 ml/g and density and viscosity 1.0014 g/ml and 0.0102 Poise for the buffer were estimated for the potassium acetate buffer using Sednterp tabulated values for sodium acetate (22). Fits were considered to be satisfactory if the rmsd was less than 0.08 and the residuals were random and <10% of the original signal.

### Thermal shift assays

This assay measures the fluorescence emission upon binding of a fluorescent probe to exposed hydrophobic regions by progressive protein denaturation with increasing temperature. The assay was performed using 5 μM purified WT full-length D6E large terminase protein in the presence of 5 mM ADP or non-hydrolysable ATP analogues and 5 mM MgCl_2_ in a 20 μl mixture containing 5 × SYPRO^®^ Orange (diluted from 5000 × stock), 5 mM HEPES pH 7.5 and 5 mM potassium glutamate, unless otherwise noted. As magnesium chloride was added in the assay buffer to facilitate nucleotide binding, its effect on protein stability was evaluated prior to the addition of ADP or non-hydrolysable ATP analogues. Prior to measurement, samples were centrifugated at 568 × *g* for 1 min to remove precipitate and bubbles. Melting curves were obtained in the temperature range of 25°C to 95°C at 1°C /min on a real-time q-PCR machine. Each condition was measured three times. Tm values were obtained by globally fitting the melting curves with a Sigmoid Function.

### Coupled ATPase assays

Coupled enzyme assays were performed at room temperature using: 6U/ml pyruvate kinase, 6U/ml lactate dehydrogenase, 1 mM phosphoenolpyruvate, 340 μM NADH, 10 mM HEPES pH 7.5, 50 mM potassium glutamate, 10 mM magnesium chloride and 0.5 mM ATP. Unless otherwise stated, reactions contained protein concentrations above the threshold for linearity: 4.5 μM full-length WT, mutant or equimolar mixtures of mutant large terminase proteins; or 0.35 μM ATPase domain. Absorbance was measured using sub-micro cell quartz cuvettes in a Cary 100 UV-visible spectrophotometer at a wave-length of 340 nm. In this assay, every NADH oxidized to NAD+ corresponds to one ATP hydrolyzed. To convert the measured absorbance directly to ATP concentration, we used the NADH extinction coefficient 6077 M^−1^cm^−1^ as measured under our experimental conditions. The ATP hydrolysis rates obtained at various ATP concentrations were fitted by the Hill equation (23) using OriginPro 2017 software.

### Crystallization and post crystallization manipulation

Crystallization was performed at 20°C by sitting drop vapour diffusion using 8 mg/ml protein solution in 20 mM Tris pH 7.5, 250 mM NaCl, 0.5 μl of protein solution was mixed with an equal volume of precipitant, before equilibrating against 100 μl of the reservoir solution. Crystals of the full-length large terminase (1–427) grew with 0.1 M HEPES pH 8.0, 1.2 M ammonium sulfate in the reservoir (Supplementary Table S1, Crystal form 1) and were cryo-protected in a solution containing 1.2 M ammonium sulfate, 0.1 M HEPES pH 8.0 and 25% (v/v) glycerol. To aid structure determination, 1 mM manganese chloride was added into the protein before crystallization. The X-ray structure, determined by multi-wavelength anomalous diffraction (MAD), showed that the ATPase domain was disordered in the crystal. Soaking these crystals in 5 M sodium chloride or 3.5 M ammonium citrate for 1 min, resulted in a new, better diffracting crystal form, where the ATPase domain was ordered (Table 1, Crystal form 2). A sulfate ion, present in the crystallization condition, was found in the ATPase active site. Initial attempts to obtain structures for complexes with ADP and non-hydrolysable ATP analogues were performed by soaking. While addition of ATP-γ-S abrogated the diffraction, in the presence of ADP, AMP-PNP or AMP-PCP, the diffraction quality was retained but no ligand was observed in the electron density. Subsequent co-crystallization trials with ADP or non-hydrolyzable ATP analogues using the WT protein resulted in crystals with the same characteristics as obtained in soaking experiments, with no ligand bound in the active site. A new construct, where the C-terminal arm residues 418–427 were deleted, was used in further trials. Crystals grew in the same conditions as the full-length protein, but were soaked in 4 M sodium formate in the presence of 50 mM ATP-γ-S or 100 mM ADP for 16 h, with 100 mM MgCl_2_ to facilitate nucleotide binding. Apo crystals were obtained using a similar procedure but with the omission of nucleotides and MgCl_2_. While the soaked crystals of this new construct retained diffraction quality, the crystal form was different to that obtained for the WT protein (Table 1, Crystal form 3).

**Table 1.** X-ray data collection and refinement statistics

Crystal form	2 HR	2* SO_4_^2−^	3	3 ATP-γ-S	3 ADP
Wavelength (Å)	0.9763	0.9763	0.9763	0.9763	0.9763
Space group	C2	C2	P1	P1	P1
Unit-cell a, b, c (Å)	180.2, 101.3, 108.5	181.3, 102.3, 110.3	100.8, 102.6, 103.1	101.4, 102.9, 103.0	100.8, 102.8, 103.0
Unit-cell α, β, γ (°)	90.0, 124.9, 90.0	90.0, 124.9, 90.0	90.7, 119.4, 117.4	90.9, 119.3, 117.1	91.1, 119.1, 117.1
Resolution(Å)	45.90–2.10 (2.14–2.10)	46.65–2.40 (2.46–2.40)	45.69–2.60 (2.65–2.60)	45.90–3.00 (3.08–3.00)	45.80–3.10 (3.19–3.10)
Rmerge (%)	7.6(123.3)	6.2(109.8)	10.3(115.3)	7.5(81.1)	16.4(83.2)
I/σ<I>	9.5(1.3)	13.6(1.2)	7.7(1.0)	6.3(1.0)	4.0 (1.0)
Completeness (%)	99.2(99.4)	99.0(94.1)	97.7(93.8)	96.5(96.5)	96.4(94.0)
Multiplicity	4.1(4.2)	4.2(3.8)	3.5(3.4)	1.9(2.0)	1.8(1.7)
CC_1/2_	0.997(0.448)	0.999(0.476)	0.995(0.331)	0.996(0.379)	0.972(0.337)
Refinement					
Rwork/Rfree	0.187/0.236	0.195/0.233	0.194/0.255	0.189/0.243	0.221/0.281
Mean B factor(A^2^)	51	73	71	79	71
R.M.S.D.					
Bond lengths (Å)	0.009	0.010	0.011	0.007	0.013
Bond angles (°)	1.4	1.3	1.5	1.1	1.7
Ramachandran (%)					
Favoured	98	98	96	95	95
Allowed	2	2	4	5	5
Outliers	0	0	0	0	0

Values in the parentheses are for the outermost resolution shell.

HR: Dataset that has the highest resolution.

*Dataset that has the fully ordered C-terminal arm.

### Data collection and structure determination

Diffraction data were collected at Diamond Light Source beamlines I02 and I04 (Table 1) and processed using XDS (24). The structure of the crystal form 1, containing a disordered ATPase domain and an ordered nuclease domain with bound Mn^2+^, was determined by MAD using SHELXD (25) and SOLVE (26), followed by density modification (RESOLVE) (27) and model building (Buccaneer) (28). Structures of crystal form 2 and 3 were determined by molecular replacement, using Phaser (29). Iterative cycles of model building and refinement were carried out using Coot (30) and REFMAC5 (31). Chimera (32) was used for figure generation.

### Molecular docking

The pentameric D6E large terminase ATPase model was generated using M-ZDOCK (33) using a similar approach as reported recently for the *Thermus* P74–26 large terminase (5). Prior to docking, two extended and potentially flexible solvent-exposed loops, residues 24–35 and 159–164, were removed, as their conformations in the monomeric and pentameric states may significantly differ.

## RESULTS

### Oligomeric state

Like T4 (34), SPP1 (35) and Sf6 (4), the full-length D6E large terminase is monomeric in solution, as judged by size exclusion chromatography (SEC) (Supplementary Figure S1A). However, subsequent analysis by sedimentation velocity analytical ultracentrifugation (SV-AUC) revealed the presence of multiple species with molecular weights corresponding to monomeric, dimeric, trimeric, tetrameric and pentameric assemblies (Supplementary Figure S1B), along with a small fraction of larger aggregates. Likewise, the ATPase domain alone was predominately monomeric but also contained a small fraction of oligomeric species (Supplementary Figure S1C) with an estimated molecular weight of ∼110 kDa, i.e. around four–five times larger than that of a single ∼27 kDa ATPase domain. These data are consistent with a pentameric oligomer state, in common with recent observations for the terminase ATPase from another thermophilic bacteriophage P74–26 (5).

### Crystal structure

Initial crystals used for structure determination by MAD, belonged to the R32 space group and contained one molecule per asymmetric unit. There was no interpretable electron density for the whole ATPase domain indicating its flexibility (Crystal form 1, Supplementary Table S1). Subsequent soaking of these crystals in cryo-protectant containing a high concentration of NaCl resulted in a transition from R32 to C2 crystal form (Crystal form 2, Table 1) with each of the ATPase domains of the three subunits related by the crystallographic 3-fold axis adopting a unique stable position. In the resulting C2 crystal form, there are three molecules in the asymmetric unit, each having a slightly different domain orientation, with an overall inter-subunit Cα rmsd of 0.8–1.6 Å (Supplementary Figure S2A).

**Figure 1.**
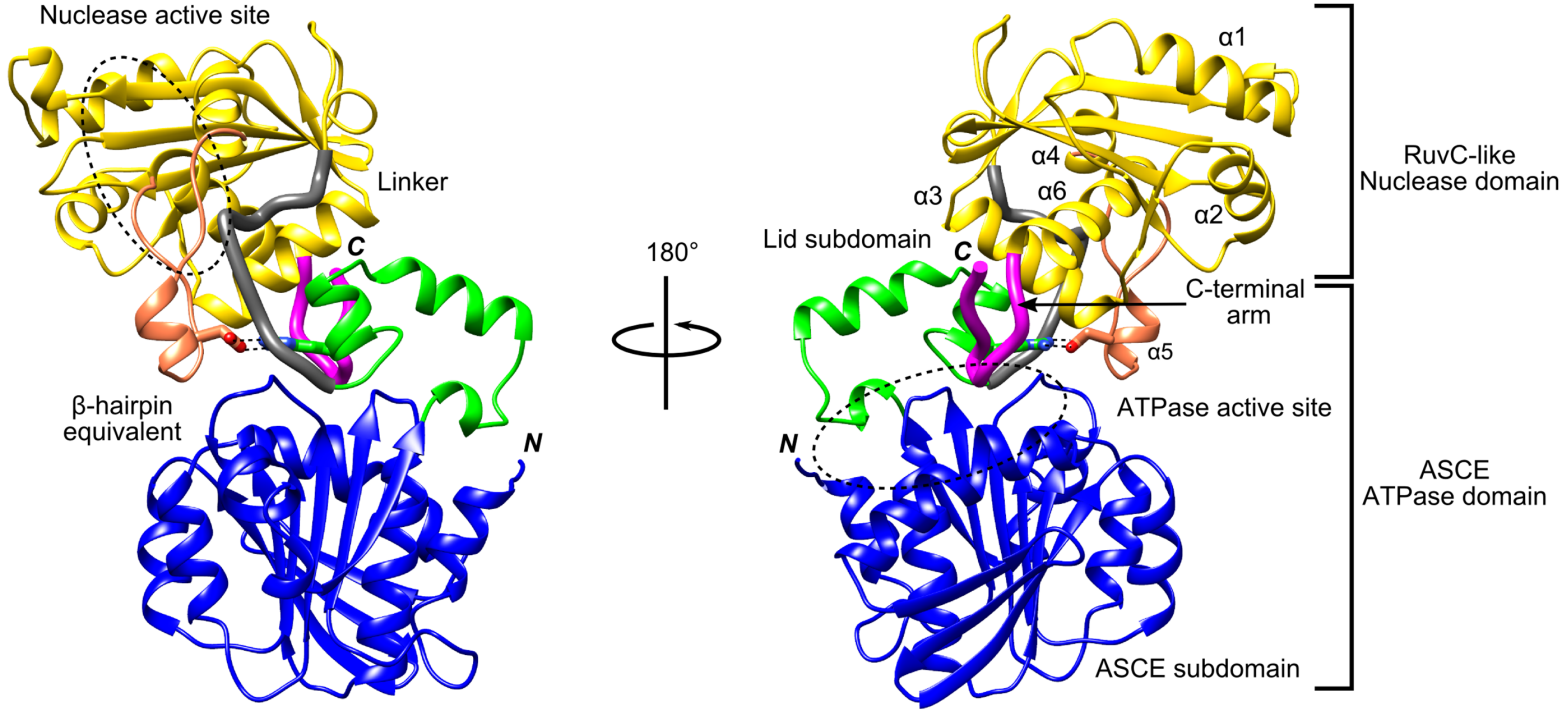
Overall structure of the D6E large terminase. Ribbon diagram showing the ATPase domain in blue (ASCE subdomain) and green (lid subdomain) and the nuclease domain in yellow. The ATPase and nuclease active sites are indicated by dashed ovals. The linker region connecting the two domain (grey) and the C-terminal arm (magenta) are shown in thicker ribbons. The extended hairpin-like structure with an α-helix at its tip, equivalent to the auxiliary β-hairpin which is present only in the large terminases family of the RNase H-like endonuclease and previously predicted to interact with DNA (12,38–40), is shown in salmon. The salt bridge between the nuclease and ATPase lid subdomain (residues D394 and R244 (in sticks) is indicated (black dashed lines)).

In common with other phages (4,5,7), the N-terminal ATPase domain of the D6E terminase comprises both an ASCE and lid subdomains facing each other (Figure 1). From the lid subdomain, the polypeptide chain leads, through an ordered linker comprising 12 amino acids (230–241), into the C-terminal nuclease domain. The nuclease domain resembles the RNase H-like fold (36,37) which, as found for the large terminases of other viruses, differs from other RNase H family proteins by an extended β-sheet and the presence of an auxiliary β-hairpin. Interestingly, the β-hairpin, previously predicted to interact with DNA (12,38–40), has an extended hairpin-like loop structure with an α-helix at its tip. The final α-helix (α6) of the nuclease domain is followed by an ordered extended C-terminal loop or ‘arm’ proximal to the ATPase active site that contains a bound sulphate ion, which was present in the crystallization condition. The relative position of the ATPase and nuclease domains is stabilized by a salt bridge formed between R224 of the lid subdomain and D394 of the hairpin-like loop, and an interdomain hydrogen bond (M216-N362; Figure 1 and Supplementary Figure S2B).

### Binding of ATP analogues

**Figure 2.**
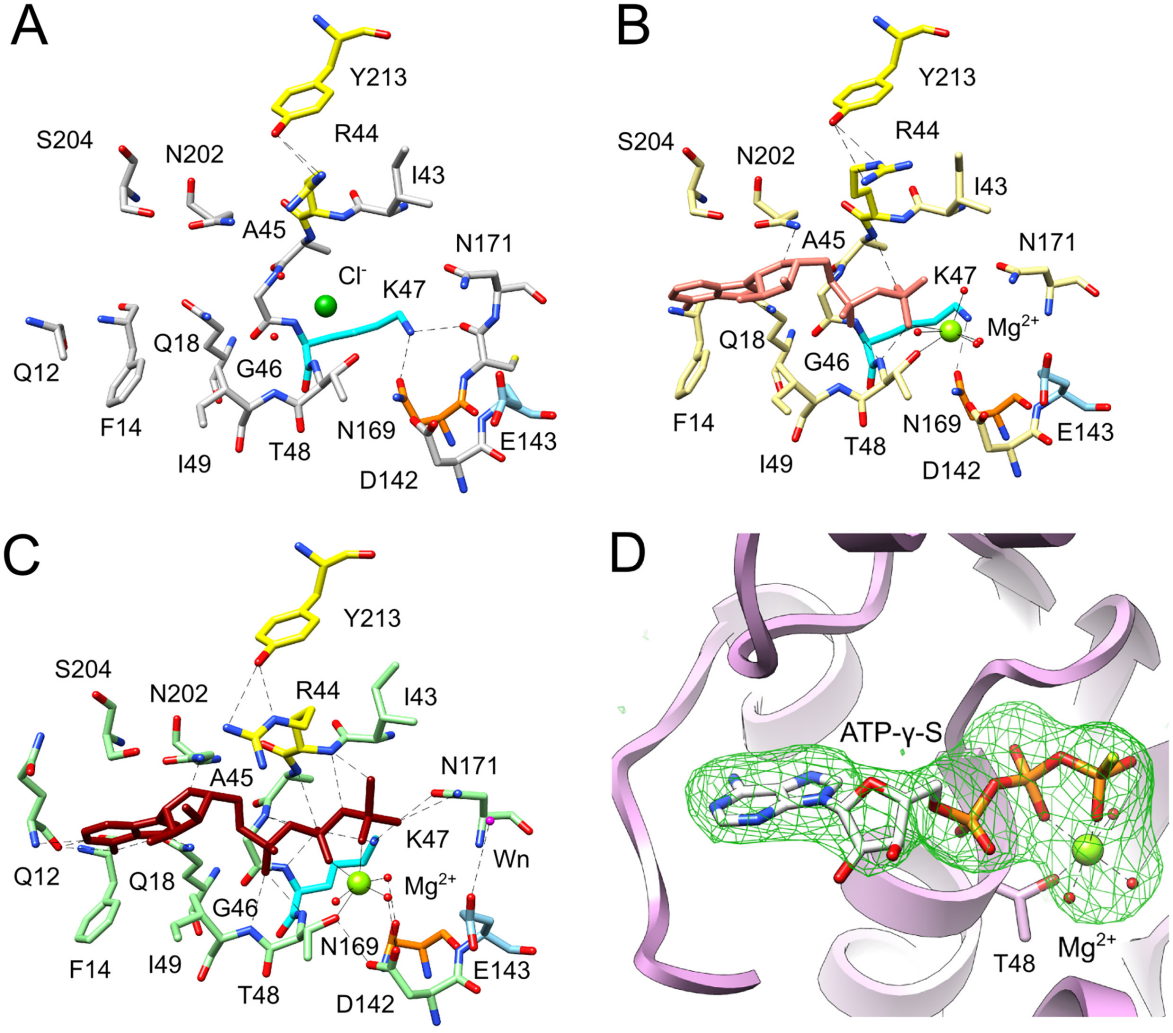
The ATPase active site. The active sites in the structures of (**A**) apo (grey carbons), (**B**) ADP complex (khaki carbons) and (**C**) ATP-γ-S complex (green carbons) are shown in ball and stick representation. The P-loop arginine R44 and the lid subdomain Y213 are highlighted in yellow, the P-loop lysine K47 in cyan and the N169 in orange. The catalytic glutamic acid expected to coordinate the catalytic water nucleophile is in blue. (**D**). The omit electron density map of the ATP-γ-S complex, contoured at 3σ level, was calculated after omitting ATP-γ-S and the coordinated metal from the model. Hydrogen bonding interactions on all diagrams are in black dashed lines, coordination to the metal (Mg^2+^, green sphere) is shown by a continuous black line.

Initial attempts to produce diffracting crystals of ligand-bound complexes of the full-length protein failed, despite thermofluor data showing stabilization of the protein in the presence of magnesium ions and either ATP-γ-S or ADP (ΔTm of 7.8 and 2.3 respectively, Supplementary Figure S3), Inspection of the structure containing bound sulphate ion in the ATPase active site showed that in crystal form 2, the C-terminal arm of the nuclease domain occludes the ATPase active site and likely requires significant reorganization to accommodate an ATP analogue (Figure 1). Consequently, a protein construct with this segment (residues 418–427) deleted was used in further crystallization trials. Structures of large terminase in complex with ATP analogues were obtained after soaking in a high salt cryo-protectant producing a different crystal form with improved diffraction quality (Crystal form 3, Table 1), as described in ‘Materials and Methods’ section. While these structures reveal very similar overall conformations, there are differences in the molecular interactions in the active site upon binding of different ATP analogues. Firstly, the side chain of R44 which hydrogen bonds to Y213 from the lid subdomain adopts different conformations upon the binding of ATP analogues (Figure 2A–D). Secondly, the hydrogen bond formed by the P-loop lysine K47 with side chains of N169 in the apo and ADP bound structures was not observed in the structure with bound ATP-γ-S (Figure 2A–D and Supplementary Figure S4). Instead, the side chain amine of this lysine forms salt bridges with the β- and γ-phosphates of the bound nucleotide. Likewise, the presence of the γ-phosphate causes N169 to rotate its side chain to hydrogen bond to an inner shell water molecule of the catalytic magnesium ion (Figure 2C). Interestingly, addition of AMP-PNP did not significantly stabilize the protein (Δ*T*_m_ = 1.4; Supplementary Figure S3) nor produced crystals with in any additional density in the active site, suggesting that the difference in its γ-phosphate conformation alters binding affinity. It is notable that pairwise comparison of three molecules taken from the asymmetric unit of the same structure reveals larger differences (Cα rmsd of 0.8–1.6 Å, Supplementary Figure S2A) than comparison of the same molecule from the asymmetric unit taken from structures of complexes with different ATP analogues (Cα rmsd of 0.3–0.4 Å; Figure S4A). This latter comparison reveals that the most significant differences occur in the lid subdomain and the P-loop (up to 1 Å Cα rmsd, Supplementary Figure S4B).

### ATP hydrolysis by the ATPase domain and full-length terminase

**Figure 3.**
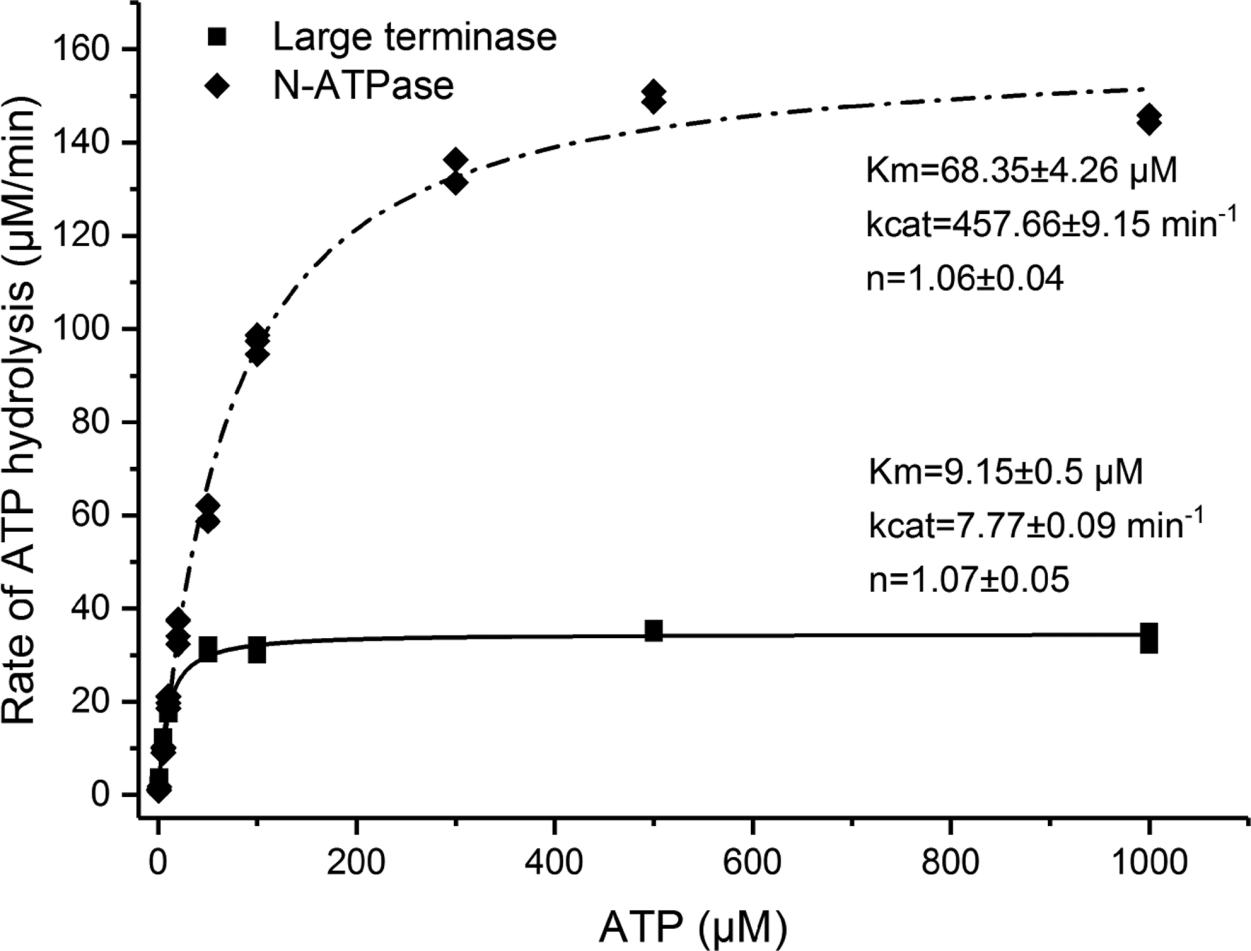
Kinetics of ATP hydrolysis. Titration of full-length large terminase (1–427) (diamonds) and the large terminase ATPase domain (1–234) (rectangles) with increasing ATP concentration. The data points (at least two repetitions each) were globally fitted with the Hill equation. *K*_m_, *k*_cat_ and the Hill constant (*n*) are indicated.

To understand the ATP hydrolysis properties of this large terminase, we compared the activities of the full-length protein with that of the ATPase domain using a steady-state ATPase activity assay (Figure 3). Measurement of ATPase activity at various protein concentrations, revealed a non-linear increase in the rate of ATP hydrolysis at low protein concentrations, indicating a concentration dependent assembly between subunits (Supplementary Figure S5). Interestingly, the ATPase domain and the full-length protein had distinct ATP binding and hydrolysis profiles (Figure 3), with the *k*_cat_ of the ATPase domain (457.7 ± 9.2 min^−1^) around 60 times higher than that of the full-length protein (7.8 ± 0.1 min^−1^). This indicates that the ATPase domain exhibits a much higher catalytic efficiency than the full-length protein. In addition, the Km of 68.4 ± 4.3 μM for this domain is approximately seven times higher than that of the full-length protein (9.2 ± 0.5 μM), suggesting a reduced level of ATP binding. Despite these differences in *k*_cat_ and *K*_m_, an apparent lack of positive cooperativity during ATP hydrolysis was observed for both proteins, with a Hill coefficient of 1.1 observed for both the full-length protein and ATPase domain (Figure 3).

### The effect of the C-terminal arm and R421 on ATP binding and hydrolysis

**Figure 4.**
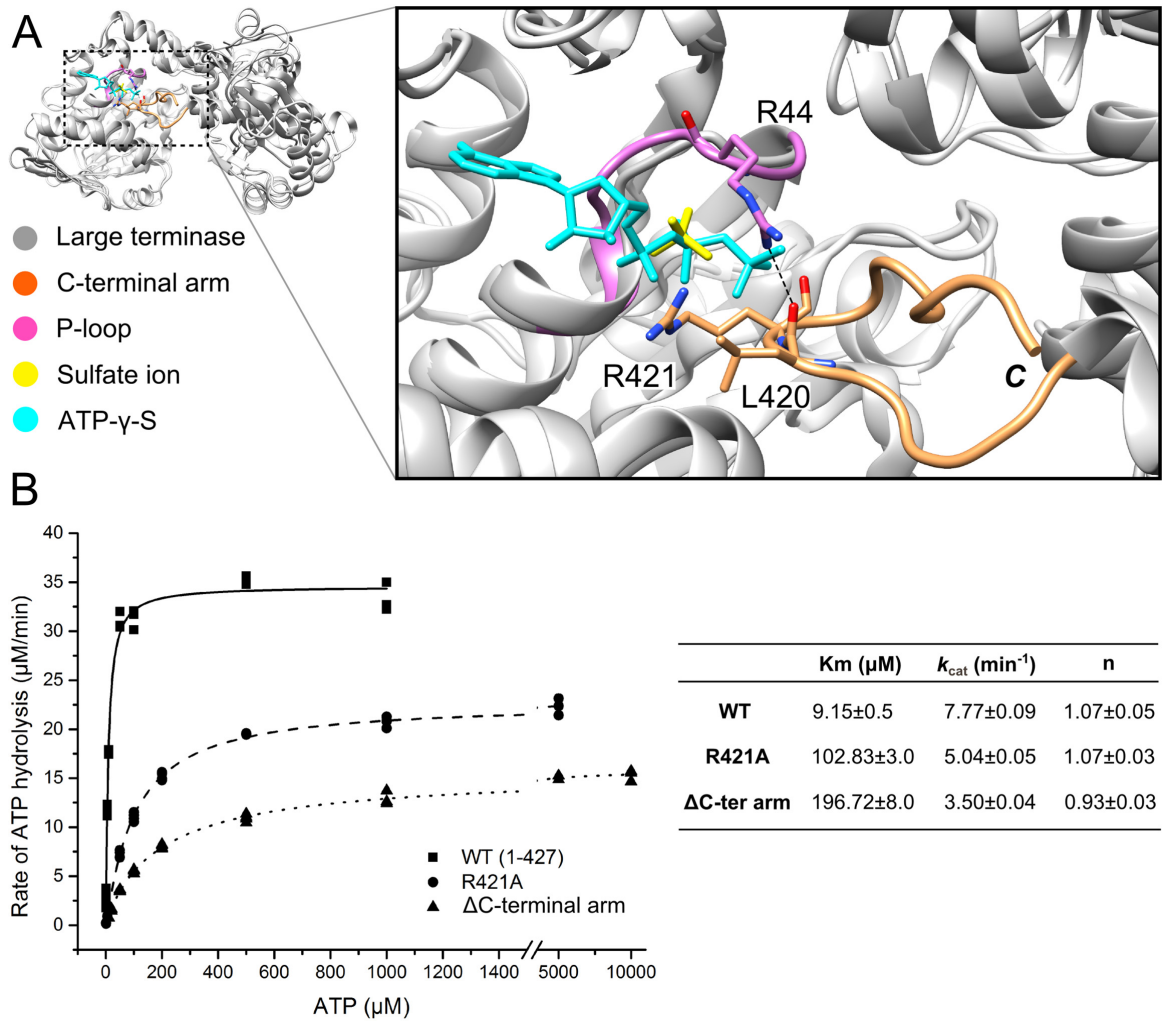
Role of the C-terminal arm. (**A**) Ribbon diagram showing structure superposition of the large terminase structures for the sulfate ion complex (white) and the ATP-γ-S complex (grey). The C-terminal arm (gold) stabilized by the sulfate ion (yellow) bound in the active site and a hydrogen bonding interaction with R44 of the P-loop (magenta) are highlighted. The interactions between the P-loop (R44) and C-terminal arm (L420) are shown in a close-up view. Hydrogen bonds are in black dashed lines. (**B**) Plot of ATP hydrolysis rate versus ATP concentration for the WT large terminase (squares), R421A mutant (circles) and C-terminally truncated protein (triangles). Data points (three repetitions each) were globally fitted with the Hill equation. *K*_m_, *k*_cat_ and Hill constant (*n*) are in the table next to panel (B).

The C-terminal arm, residues 418–427 of the nuclease domain, lies close to the ATPase active site (Figure 1) and thus may play a role in ATP binding and/or hydrolysis. Indeed, closer analysis reveals that this ordered extended turn contributes to the active site (Figures 1, 4A and Supplementary Figure S4A) through an arginine residue (R421) at the tip of the C-terminal arm, that is in proximity to the sulphate ion bound in the active site. Additionally, the carbonyl oxygen of the preceding residue, L420, formed a hydrogen bonding interaction with R44 of the P-loop of the lid subdomain (Figure 4A). Superposition of the structures for the full-length protein and the truncated protein (with bound sulphate or ATP-γ-S, respectively) positions R421 close to the α-phosphate of the ATP-γ-S (Figure 4A). However, the fact that the presence of this C-terminal arm was incompatible with the formation of well-ordered, diffracting crystals of ATP analogue-bound complexes suggests conformational plasticity. It is therefore possible that this residue may interact with β- or γ- but not necessarily the α-phosphate. To further investigate the role of the C-terminal arm, and R421 in particular, on ATP binding and hydrolysis, we determined the *K*_m_ and *k*_cat_ values for the C-terminally truncated protein (1–417) and the full-length R421A mutant. Compared to the WT large terminase (Figures 3 and 4B), both mutants had reduced ATP binding affinity, as defined by the observed increase in the *K*_m_ values of ∼10- and 20-fold, for the R421A mutant and the C-terminally truncated protein, respectively (Figure 4B). Despite the drastic change in the *K*_m_, the catalytic efficiency (*k*_cat_) showed only a slight decrease compared to the WT protein. These data collectively suggest that the C-terminal arm plays a role in ATP binding, potentially serving to stabilize the lid subdomain and the active site in a conformation compatible with ATP binding in the monomeric form.

### A model for D6E ATPase motor

**Figure 5.**
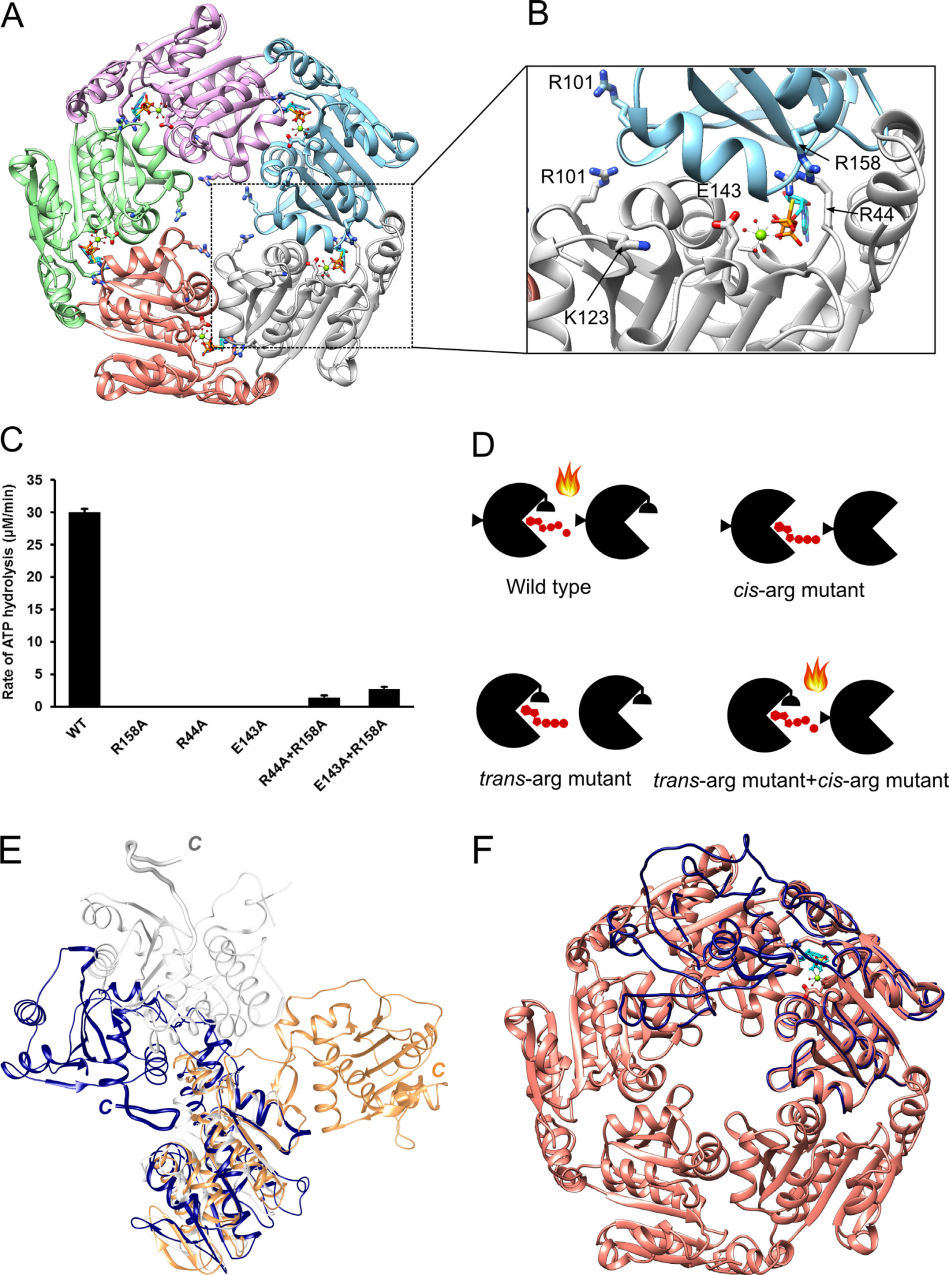
Model for pentamer assembly and identification of the *trans*-arginine finger. (**A**) Pentameric molecular docking model of the D6E large terminase ATPase. (**B**) A close-up view of an ATPase active site at the interface between two subunits. ATP-γ-S (cyan/orange) is shown in sticks. The side chain of R101, K123, R44, E143 and R158 are shown in thick sticks. (**C**) Steady-state ATPase activity of WT, R44A, E143A, R158A, equal molar mixtures of R158 to E143A or R44A. (**D**) Schematic representations of the complementary ATPase assays. The ATPase subunit is represented by an open circle with the active site located at the subunit interface. The *cis*- and *trans-*arginines are shown as semi-circles and triangles, respectively. A mixture of *trans-* and *cis*- arginine mutants leads to the formation of an intact active site able to hydrolyze ATP (represented by the flame). (**E**) Superposition of the ATPase domain of D6E (navy blue), T4 (coral) and Sf6 (white) large terminases. (**F**) Structure superposition of the ATPase domain of full-length D6E large terminase (navy blue) onto a subunit of the pentameric molecular docking model of the ATPase ring (salmon).

**Figure 6.**
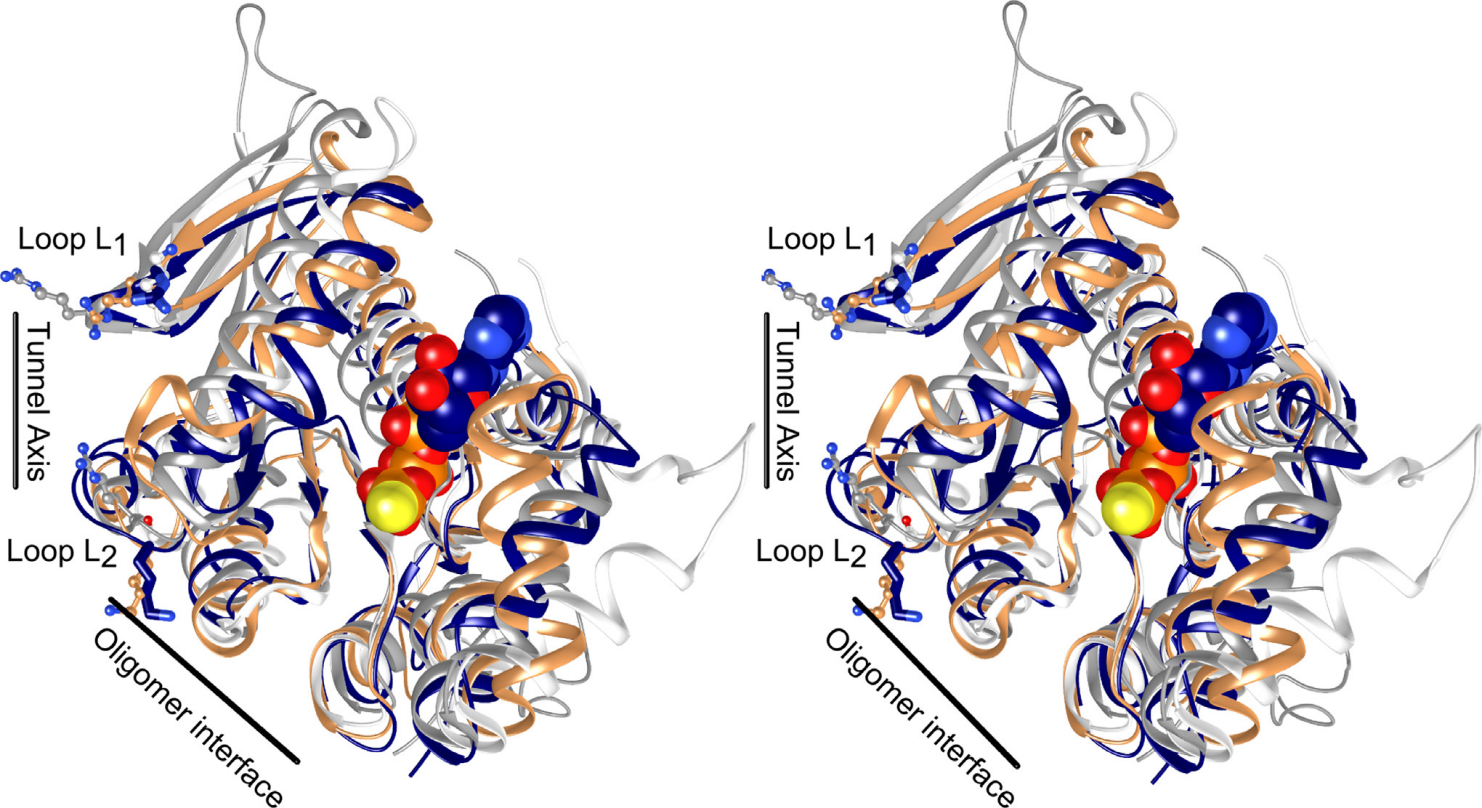
Comparison of DNA tunnel loops and oligomer interface. Structure superposition, shown in stereo, of D6E (navy blue), T4 (white), Sf6 (coral) and P74–26 (grey) large terminase ATPases. The bound ATP-γ-S is shown in spheres. The tunnel axis and oligomer interface are indicated using black lines. The position of L_1_ and L_2_ are indicated. R101 and K123 for the D6E large terminase are shown in thick sticks. Their equivalent residues in T4, Sf6 and P74–26 are shown in ball and sticks.

To gain understanding of how the motor is assembled, we constructed a pentamer model of the D6E ATPase motor by molecular docking (33). The resulting model of the pentamer contains a central tunnel of ∼18 Å in diameter, with the ATPase active sites found at monomer-monomer interfaces (Figure 5A), in common with the pentamer models for phi29 (16) and P74–26 (5). In the pentamer, the bound nucleotide is in proximity to R44 and E143 from one subunit and R158 from the adjacent subunit (Figure 5B). Notably, two loops defined as L_1_ and L_2_, each containing a positively charged residue, R101 and K123, respectively (Figure 5B), are found to contribute to the central tunnel and are expected to interact with DNA. We note that equivalent positively charged residues are also present in P74–26 (confirmed by mutagenesis to be required for DNA binding (5)) and Sf6 large terminases (Figure 6). In common with models of large terminase ATPases from other viruses and structural observations for hexameric helicases (5,16,41,42), the tunnel surface is mostly positively charged (Supplementary Figure S6).

### Identification of the *trans* arginine finger

Previous sequence analysis of the HerA/FtsK superfamily of ASCE ATPases revealed a conserved arginine, indicative of ring-like oligomeric ATPases, likely to promote inter-subunit coordination of ATP hydrolysis in *trans* (43,44). Recent structural and biochemical studies of the phi29 packaging ATPase and P74–26 large terminases identified the *trans*-arginine residues R146 and R139, respectively, located at the monomer-monomer interfaces of the corresponding pentamer models (5,16). R158, which projects into the active site from the adjacent subunit in our pentamer model (Figure 5B), is predicted to stabilize the transition state and facilitate ATP hydrolysis *in trans*. Additional active site residues such as the P-loop arginine, or ‘sensor’, and the catalytic glutamate were identified in other large terminases as facilitating ATP catalysis *in cis* (Figure 5B) (3–5). The equivalent three residues of the D6E large terminase, respectively, predicted to be R158 (*trans*), R44 and E143 (*cis*), should be indispensable for catalysis. We tested this by mutagenesis, showing that substitution of any one of these residues to alanine completely abrogated ATP hydrolysis (Figure 5C). However, this activity was partially recovered in assays where R158A terminase was complemented with either R44A or E143A proteins (Figure 5C), as previously found for the P74–26 large terminase (5) and other ring-like ATPase motors (16,45). These data are consistent with the pentamer model since complementation requires oligomer assembly to create a catalytically competent active site at the subunit interface containing both *trans*- and *cis-* arginines. These data confirmed that R158 is indeed the *trans* residue for D6E (Figure 5C and D).

## DISCUSSION

We here provide data from a combination of biophysical, biochemical, structural and molecular docking experiments, which, together support a model where the constituent domains of the D6E large terminase protein undergo radical rearrangement to both enable, and regulate, assembly into a functional pentameric motor capable of ATP hydrolysis and DNA translocation.

### ATP binding and hydrolysis

#### The trans-arginine

In multi-subunit ASCE ATPases, a *trans-*arginine finger residue is generally required to couple ATP hydrolysis between the subunits of the active ring-like assembly (10,45,46). Comparative genomic analysis has indicated that although the presence of the *trans*-arginine finger is commonly found for multi-subunit ASCE ATPases, the position of this finger varies across different superfamilies (9,10). Within the HerA/FtsK superfamily, conserved arginine residues identified between α3 and β4 of the canonical ASCE fold have been proposed to be the *trans*-arginine finger (10). The biochemically confirmed *trans-*arginine residues, R158, and R146 for D6E (Figure 5A–C) and phi29 packaging ATPases (16,47), respectively, are found at the equivalent position on the overall fold coinciding with the position of the *trans-*arginine in the HerA/FtsK superfamily (Supplementary Figure S7). Arginine residues are also found in corresponding positions for Sf6, T4 and P74–26 large terminase proteins (4,5,48) but have not been confirmed in activity assays (Supplementary Figure S7). Conversely, the biochemically confirmed *trans*-arginine finger, R139, for the P74–26 large terminase is located between α2 and β3 (Supplementary Figure S7) where an arginine residue is also found for T4. We note that a comparable lysine at this position could also potentially act as the trans-acting finger for Sf6. Further biochemical mutation complementation assays are required to identify the *trans*-acting residue for Sf6 and T4 large terminases. Despite this, our study supports the premise that large terminase proteins are indeed multi-subunit ring-like ATPases, with a conserved *trans*-arginine finger that complements the ATPase active site at the subunit interface. These data also suggest that large terminase ATPases may be closely related to the HerA/FtsK superfamily, as previously proposed for the phi29 packaging ATPase (10). Furthermore, pentamer assemblies were observed for both full-length protein and the isolated ATPase domain, by SV-AUC and SEC respectively. This, along with the biochemical and structural confirmation of a conserved *trans*-arginine finger in this, and other, bacteriophage large terminases, further supports the notion that DNA translocation is driven by inter-subunit coordination of ATP hydrolysis (5).

#### Cis residues

The P-loop arginine found in many bacteriophage large terminases and packaging ATPases, including T4, Sf6, P74–26, λ and phi29 (4,5,16,48,49) and other multi-subunit ATPases (50,51), corresponds to R44 in the D6E large terminase (Figure 5A and B). It has been proposed that this arginine may act in*cis* to trigger ATP hydrolysis through subdomain rotation and/or chemo-mechanical coupling to coordinate DNA translocation (3–5). Recent studies for λ large terminase support the proposed *cis* mechanism implicated previously, but further suggests that it is likely to play a role in motor assembly (52). Structural and biochemical observations on the D6E large terminase are consistent with R44 being a *cis* arginine that couples the motion of the lid subdomain to ATP hydrolysis. However, similar to the observation for Sf6 large terminase (4), the observed overall conformational differences upon nucleotide binding are insignificant. It is likely that larger conformational changes that drive DNA translocation are dependent on the presence of other motor components and require the participation of the *trans*-arginine finger from the adjacent subunit within the pentameric assembly.

Our results provide insight into the mechanism of ATP hydrolysis of the large terminase motor. In the assembled motor, R158 facilitates ATP hydrolysis *in trans* while R44 acts *in cis* to transmit the conformational changes of the P-loop upon ATP hydrolysis through the lid subdomain.

### Motor assembly and regulation

Formation of the D6E pentameric assembly likely requires large rearrangements in relative domain orientation. Such flexibility is implied from superposition of the ATPase domain of the T4, Sf6 and D6E large terminase structures revealing that a significant rotation is required to move the D6E nuclease domain in order for it to occupy a similar position to that observed in T4 or Sf6 (Figure 5E). This is also consistent with the observation that the domain orientation seen in the crystal structure is incompatible with our proposed pentameric ATPase model (Figure 5F), with the nuclease domain clashing with the ATPase of the adjacent subunit, and the fact that the ATPase domain alone is significantly more efficient at hydrolyzing ATP than the WT large terminase (∼50-fold increase in *k*_cat_ Figure 3). This can also explain the concentration-dependent pentamer assembly observed, for the full-length protein, in SV-AUC (undiluted), but not by SEC where the protein concentration was diluted during chromatography (53) (Supplementary Figure S1). This observation is also supported by the non-linearity observed in the concentration-dependent ATPase rate (Supplementary Figure S5); however, further structural studies of the assembled motor or its intermediates, along with single molecule FRET studies are required to confirm this. Consistent with previous studies (5), only modest or low positive cooperativity for ATP hydrolysis (Hill coefficient 1.1 for D6E and 1.7 for P74–26) was observed. This suggests that, in the absence of DNA and/or the portal ring, the motor may be a partially assembled pentamer, where only 1–2 functional ATPase active site(s) are formed by 2–3 subunits. This premise is consistent with the observation of intermediate oligomeric states by SV-AUC. Alternatively, as proposed for the phi29 DNA packaging motor (Hill constant 1.2 measured by ATPase assays during DNA packaging) (54), binding of ATP to a monomer in the fully assembled pentameric motor is independent of its binding to the adjacent subunits (55).

In the assembled pentamer, loops L_1_ and L_2_ are predicted to project into the tunnel where they may engage in DNA translocation. This is supported by structural superpositions with super family 1 helicase UrvD (56) (the closest structural homologue identified by a DALI search, Z-score = 13.1 (57)) and the hexameric helicase E1 (42) (Supplementary Figure S8). Mutagenesis and DNA binding studies are required to confirm if positively charged residues found on these loops do indeed contribute to large terminase-DNA interaction. Loop L_2_ precedes an α-helix (residues 125–132) that adopts somewhat different positions within each molecule of an asymmetric unit (Supplementary Figure S2A) and this conformational flexibility may to be required for DNA binding or translocation.

**Figure 7.**
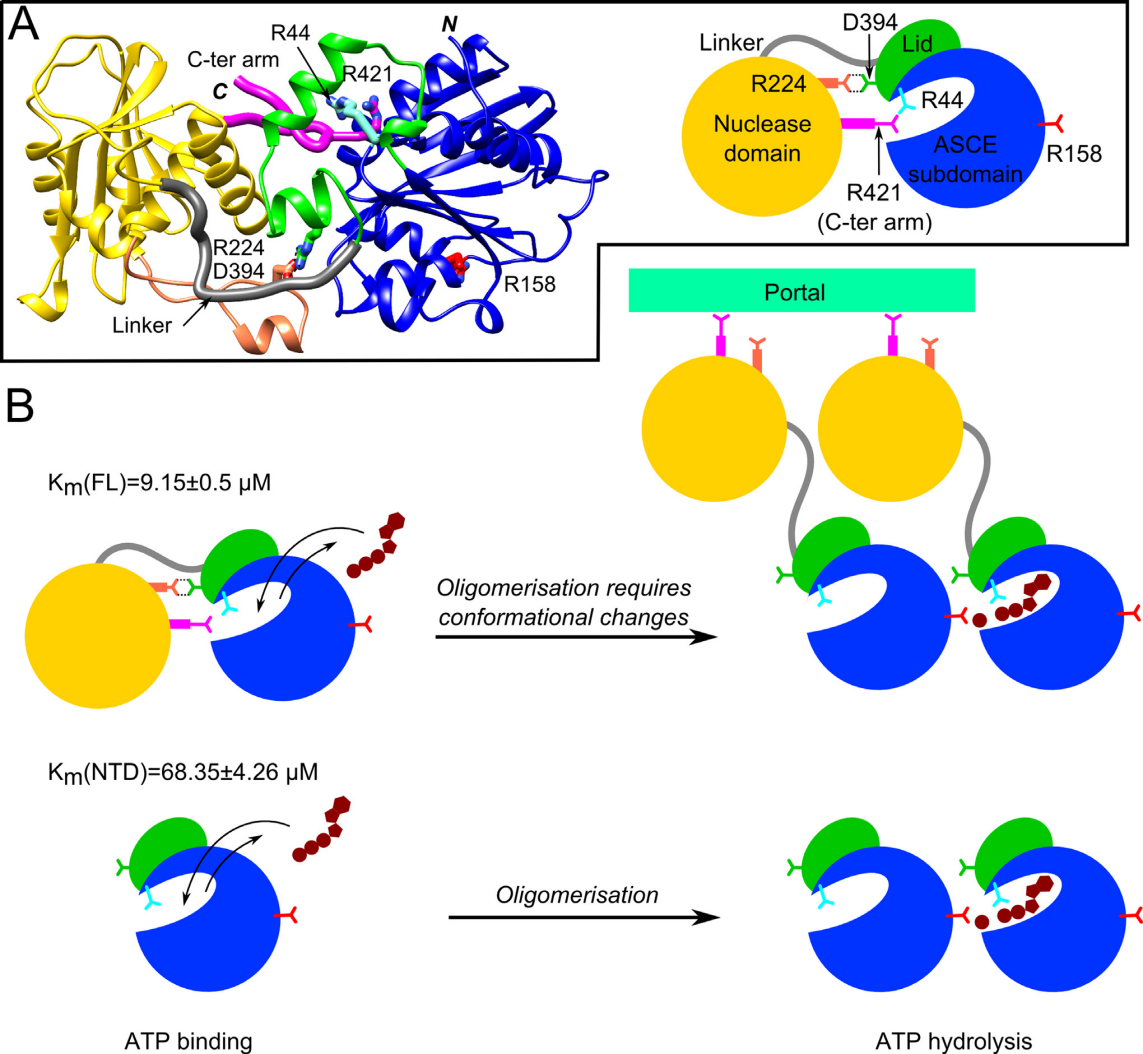
Proposed mechanism for large terminase motor assembly. (**A**) Schematic representations of the D6E large terminase. The ATPase and nuclease domain of the D6E large terminase protein are represented by coloured spheres. Structural elements that are involved in the proposed mechanism are shown in corresponding colours used in Figure 1. The side chain of R44 (cyan), R158 (fire brick) and R421 (magenta) are shown in sticks. (**B**) Schematic model for large terminase motor assembly and coupled ATP hydrolysis. The scheme describes the proposed mechanism of ATP binding, hydrolysis and oligomerization for the full-length large terminase (top) and ATPase domain alone (bottom).

ATP bound in the monomer active site cannot be hydrolyzed without the participation of the *trans*- arginine finger from an adjacent subunit in the assembled pentamer (Figure 7A and B), which requires significant rotation of the nuclease domain with respect to the ATPase domain. This monomer conformation is stabilized, or ‘locked’, by the C-terminal arm. There are also additional ATPase-nuclease domain–domain interactions (Figure 1, Supplementary Figure S2, Tables S2 and 3) resulting in a total surface area buried between the two domains of 362 Å^2^. For comparison, areas buried in inter-domain interactions in T4 and Sf6, are significantly smaller, 212 and 78 Å^2^, respectively. It is possible the distinct domain arrangement of the D6E large terminase stabilized by these interactions represents a biologically important conformationally locked inactive ‘initiation’ state that serves to prevent futile hydrolysis of ATP before assembly of this extremophilic motor.

It is possible that oligomerization of the large terminase into a stable pentameric motor capable of DNA translocation may only occur after interaction with the portal protein as this may stabilize the domain reorientation (Figure 7A and B) which would be required for the pentamer assembly. Since the C-terminal segment of the nuclease domain is thought to interact with portal (58), we speculate that the disengagement of the nuclease domain from its own ATPase frees this segment to interact with the portal assembly, enabling neighbouring large terminase subunits to form monomer–monomer interactions that may further inhibit the restoration of the ‘initiation’ state. Combined with the increased local concentration of large-terminase proteins at the portal vertex of the capsid, these processes might assist in overcoming the rate limiting step of a co-ordinated interdomain conformational change and oligomerization of the ATPase subunits (Figure 7A and B), thus allowing more efficient assembly and ATP hydrolysis (Figure 3; Supplementary Figures S1 and 5).

### The structure of the C-terminal arm and its potential regulatory role

**Figure 8.**
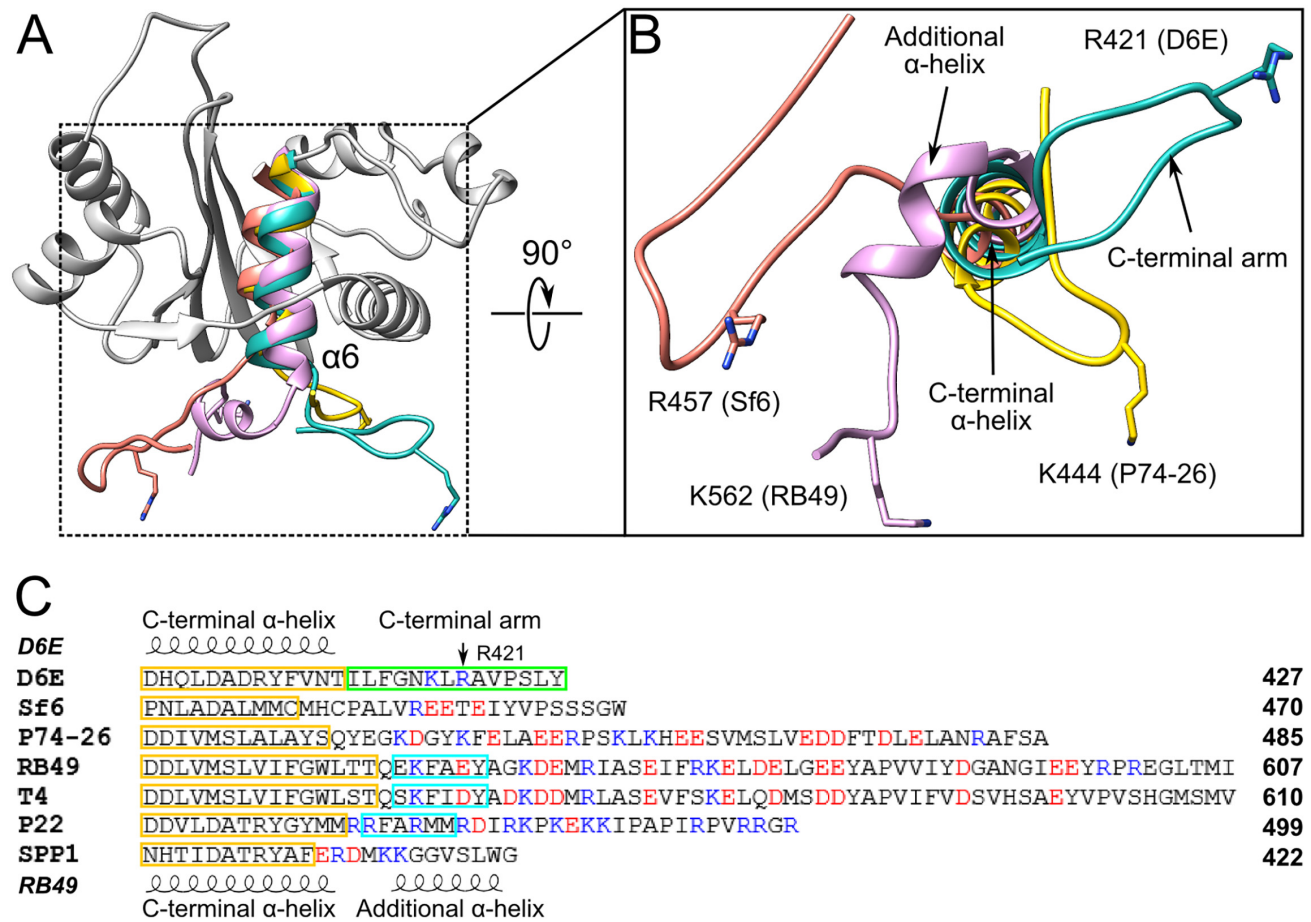
Comparison of C-terminal arms. (**A**) Structure superposition of D6E (grey/green), Sf6 (salmon), RB49 (light pink) and P74–26 (yellow) large terminase nuclease domains. For clarity, only the C-terminal α-helix (α6) is shown in the structural superposition, except for D6E where the nuclease domain structure is shown. (**B**) A close-up view of the C-terminal arm of the D6E large terminase and equivalent structural elements observed in the structures of Sf6, RB49 and P74–26 large terminases. Positively charged residues found on the tip to the arm are shown in sticks. (**C**) Protein sequence comparison of the C-terminal region of D6E with Sf6, P74–26, RB49, T4 and P22 large terminases. The secondary structure of D6E and RB49 are shown on the top and bottom of the sequence alignment. The C-terminal α-helix is outlined in yellow. The position of R421 and C-terminal arm (green box) of the D6E large terminase are indicated. The additional C-terminal α-helix only found in RB49, T4 and P22 large terminases are highlighted (cyan boxes). Positively and negatively charge residues are coloured in blue and red respectively.

Our structural and biochemical data show that a short segment after the C-terminal α-helix (α6 in Figure 8) of the nuclease domain, the C-terminal arm, plays a role in stabilizing the ATP bound state in the monomeric form of D6E. Indeed, these data suggest that this effect is mostly due to R421, predicted to contact a phosphate group of the ligand and biochemically confirmed by mutagenesis to decrease ATP binding (increased *K*_m_, Figure 4B). This may play a similar role to the P-loop residue, R44, by facilitating the binding of the tri-phosphate in*cis*. Interestingly, similar ordered C-terminal arm elements, displaying clusters of either negatively charged or hydrophobic residues around a positive amino acid (K or R) close to the turn, are found in the large terminase proteins from P74–26, Sf6 and RB49 bacteriophages (Figure 8). For the nuclease structures of bacteriophages T4 and P22, where the C-terminal segments were mostly disordered, alignment of the C-terminal regions also reveal conserved positively charged residues (Figure 8). Indeed, the analogous residue to D6E R421 in T4 phage (K567) lies at the end of a negatively charged cluster of residues, near to S583, respectively proposed- and proven- to be involved in portal protein interaction (59). The observations that this structural element is conserved indicate that it plays a biologically important role in motor function, most likely mediating the assembly of large terminase onto the portal protein embedded in the preformed capsid. Further work will determine if, as observed for the D6E large terminase, ATP binding to the monomeric state (prior to motor assembly) facilitated by the C-terminal arm also occurs in other phage systems. Additionally, this structural element may also serve to restrict assembly of the pentameric motor, and subsequent futile ATP hydrolysis, until procapsid formation.

We here provide evidence that the D6E bacteriophage large terminase protein is a pentameric ring-like ATPases related to the HerA/FtsK superfamily utilizing a similar *trans*-arginine finger to trigger ATP hydrolysis. Additionally, we identify a new structural element, the C-terminal arm of the nuclease domain, as a potentially conserved feature of large terminase proteins that is implicated in the assembly of active motor complex onto the portal vertex of viral capsid. The combination of crystallographic and biochemical data indicate that the arm may play a role in both regulating and coupling the hydrolysis of pre-bound ATP with the motor assembly events. Future work is required to verify this model for other large terminases and to ascertain the residues that are important for ATP binding, motor assembly, and for coupling ATP hydrolysis events with DNA translocation.

## AVAILABILITY

Structures of the D6E large terminase have been deposited with the Protein Data Bank, accession codes 5OE8 (Crystal form 2), 5OE9 (Crystal form 2, SO_4_^2−^), 5OEA (Crystal form 3, ATP-γ-S), 5OEB (Crystal form 3, ADP), 5OEE (Crystal form 3).

## Supplementary Material

Supplementary Data

## References

[1] Casjens S.R. The DNA-packaging nanomotor of tailed bacteriophages. Nat. Rev. Microbiol. 2011; 9:647–657. 21836625 10.1038/nrmicro2632

[2] Feiss M., Rao V. The bacteriophage packaging machine. Adv. Exp. Med. Bio. 2012; 726:489–509. 22297528 10.1007/978-1-4614-0980-9_22

[3] Sun S., Kondabagil K., Draper B., Alam T.I., Bowman V.D., Zhang Z., Hegde S., Fokine A., Rossmann M.G., Rao V.B. The structure of the phage T4 DNA packaging motor suggests a mechanism dependent on electrostatic forces. Cell. 2008; 135:1251–1262. 19109896 10.1016/j.cell.2008.11.015 PMC12969755

[4] Zhao H., Christensen T.E., Kamau Y.N., Tang L. Structures of the phage Sf6 large terminase provide new insights into DNA translocation and cleavage. Proc. Natl. Acad. Sci. U.S.A. 2013; 110:8075–8080. 23630261 10.1073/pnas.1301133110 PMC3657791

[5] Hilbert B.J., Hayes J.A., Stone N.P., Duffy C.M., Sankaran B., Kelch B.A. Structure and mechanism of the ATPase that powers viral genome packaging. Proc. Natl. Acad. Sci. U.S.A. 2015; 112:E3792–E3799. 26150523 10.1073/pnas.1506951112 PMC4517215

[6] Alam T.I., Rao V.B. The ATPase domain of the large terminase protein, gp17, from bacteriophage T4 binds DNA: implications to the DNA packaging mechanism. J. Mol. Biol. 2008; 376:1272–1281. 18234214 10.1016/j.jmb.2007.12.041

[7] Sun S., Kondabagil K., Gentz P.M., Rossmann M.G., Rao V.B. The structure of the ATPase that powers DNA packaging into bacteriophage T4 procapsids. Mol. Cell. 2007; 25:943–949. 17386269 10.1016/j.molcel.2007.02.013

[8] Rao V.B., Mitchell M.S. The N-terminal ATPase site in the large terminase protein gp17 is critically required for DNA packaging in bacteriophage T4. J. Mol. Biol. 2001; 314:401–411. 11846554 10.1006/jmbi.2001.5169

[9] Burroughs A., Iyer L., Aravind L. Volff J-N The bacteriophage packaging machine. Gene and Protein Evolution. 2007; 3:Basel: Karger Publishers 48–65.

[10] Iyer L.M., Makarova K.S., Koonin E.V., Aravind L. Comparative genomics of the FtsK–HerA superfamily of pumping ATPases: implications for the origins of chromosome segregation, cell division and viral capsid packaging. Nucleic Acids Res. 2004; 32:5260–5279. 15466593 10.1093/nar/gkh828 PMC521647

[11] Black L.W. DNA packaging in dsDNA bacteriophages. Annu. Rev. Microbiol. 1989; 43:267–292. 2679356 10.1146/annurev.mi.43.100189.001411

[12] Xu R.-G., Jenkins H.T., Chechik M., Blagova E.V., Lopatina A., Klimuk E., Minakhin L., Severinov K., Greive S.J., Antson A.A. Viral genome packaging terminase cleaves DNA using the canonical RuvC-like two-metal catalysis mechanism. Nucleic Acids Res. 2017; 45:3580–3590. 28100693 10.1093/nar/gkw1354 PMC5389553

[13] Majorek K.A., Dunin-Horkawicz S., Steczkiewicz K., Muszewska A., Nowotny M., Ginalski K., Bujnicki J.M. The RNase H-like superfamily: new members, comparative structural analysis and evolutionary classification. Nucleic Acids Res. 2014; 42:4160–4179. 24464998 10.1093/nar/gkt1414 PMC3985635

[14] Ponchon L., Boulanger P., Labesse G., Letellier L. The Endonuclease Domain of Bacteriophage Terminases Belongs to the Resolvase/Integrase/Ribonuclease H Superfamily A bioinformatics analysis validated by a functional study on bacteriophage T5. J. Biol. Chem. 2006; 281:5829–5836. 16377618 10.1074/jbc.M511817200

[15] Ye J., Osborne A.R., Groll M., Rapoport T.A. RecA-like motor ATPases—lessons from structures. Biochim. Biophys. Acta. 2004; 1659:1–18. 15511523 10.1016/j.bbabio.2004.06.003

[16] Mao H., Saha M., Reyes-Aldrete E., Sherman M.B., Woodson M., Atz R., Grimes S., Jardine P.J., Morais M.C. Structural and molecular basis for coordination in a viral DNA packaging motor. Cell Rep. 2016; 14:2017–2029. 26904950 10.1016/j.celrep.2016.01.058 PMC4824181

[17] Daudén M.I., Martín-Benito J., Sánchez-Ferrero J.C., Pulido-Cid M., Valpuesta J.M., Carrascosa J.L. Large terminase conformational change induced by connector binding in bacteriophage T7. J. Biol. Chem. 2013; 288:16998–17007. 23632014 10.1074/jbc.M112.448951 PMC3675631

[18] Massey T.H., Mercogliano C.P., Yates J., Sherratt D.J., Löwe J. Double-stranded DNA translocation: structure and mechanism of hexameric FtsK. Mol. Cell. 2006; 23:457–469. 16916635 10.1016/j.molcel.2006.06.019

[19] Wang Y., Zhang X. Genome analysis of deep-sea thermophilic phage D6E. Appl. Environ. Microbiol. 2010; 76:7861–7866. 20889772 10.1128/AEM.01270-10 PMC2988599

[20] Fogg M.J., Wilkinson A.J. Higher-throughput approaches to crystallization and crystal structure determination. Biochem. Soc. Trans. 2008; 36:771–775. 18631156 10.1042/BST0360771

[21] Brown P.H., Schuck P. A new adaptive grid-size algorithm for the simulation of sedimentation velocity profiles in analytical ultracentrifugation. Comput. Phys. Commun. 2008; 178:105–120. 18196178 10.1016/j.cpc.2007.08.012 PMC2267755

[22] Philo J., Hayes D., Laue T. Sednterp. 2006; Thousand Oaks: Alliance Protein Laboratories.

[23] Weiss J.N. The Hill equation revisited: uses and misuses. FASEB J. 1997; 11:835–841. 9285481

[24] Kabsch W. XDS. Acta Crystallogr. D Biol. Crystallogr. 2010; 66:125–132. 20124692 10.1107/S0907444909047337 PMC2815665

[25] Schneider T.R., Sheldrick G.M. Substructure solution with SHELXD. Acta Crystallogr. D Biol. Crystallogr. 2002; 58:1772–1779. 12351820 10.1107/s0907444902011678

[26] Terwilliger T.C., Berendzen J. Automated MAD and MIR structure solution. Acta Crystallogr. D Biol. Crystallogr. 1999; 55:849–861. 10089316 10.1107/S0907444999000839 PMC2746121

[27] Terwilliger T.C. Maximum-likelihood density modification. Acta Crystallogr. D Biol. Crystallogr. 2000; 56:965–972. 10944333 10.1107/S0907444900005072 PMC2792768

[28] Cowtan K. The Buccaneer software for automated model building. 1. Tracing protein chains. Acta Crystallogr. D Biol. Crystallogr. 2006; 62:1002–1011. 16929101 10.1107/S0907444906022116

[29] McCoy A.J., Grosse-Kunstleve R.W., Adams P.D., Winn M.D., Storoni L.C., Read R.J. Phaser crystallographic software. J. Appl. Crystallogr. 2007; 40:658–674. 19461840 10.1107/S0021889807021206 PMC2483472

[30] Emsley P., Cowtan K. Coot: model-building tools for molecular graphics. Acta Crystallogr. D Biol. Crystallogr. 2004; 60:2126–2132. 15572765 10.1107/S0907444904019158

[31] Murshudov G.N., Vagin A.A., Dodson E.J. Refinement of macromolecular structures by the maximum-likelihood method. Acta Crystallogr. D Biol. Crystallogr. 1997; 53:240–255. 15299926 10.1107/S0907444996012255

[32] Pettersen E.F., Goddard T.D., Huang C.C., Couch G.S., Greenblatt D.M., Meng E.C., Ferrin T.E. UCSF Chimera—a visualization system for exploratory research and analysis. J. Comput. Chem. 2004; 25:1605–1612. 15264254 10.1002/jcc.20084

[33] Pierce B., Tong W., Weng Z. M-ZDOCK: a grid-based approach for C n symmetric multimer docking. Bioinformatics. 2004; 21:1472–1478. 15613396 10.1093/bioinformatics/bti229

[34] Leffers G., Rao V.B. Biochemical characterization of an ATPase activity associated with the large packaging subunit gp17 from bacteriophage T4. J. Biol. Chem. 2000; 275:37127–37136. 10967092 10.1074/jbc.M003357200

[35] Gual A., Camacho A.G., Alonso J.C. Functional analysis of the terminase large subunit, G2P, of Bacillus subtilis bacteriophage SPP1. J. Biol. Chem. 2000; 275:35311–35319. 10930407 10.1074/jbc.M004309200

[36] Górecka K.M., Komorowska W., Nowotny M. Crystal structure of RuvC resolvase in complex with Holliday junction substrate. Nucleic Acids Res. 2013; 41:9945–9955. 23980027 10.1093/nar/gkt769 PMC3834835

[37] Ariyoshi M., Vassylyev D.G., Iwasaki H., Nakamura H., Shinagawa H., Morikawa K. Atomic structure of the RuvC resolvase: a Holliday junction-specific endonuclease from E. coli. Cell. 1994; 78:1063–1072. 7923356 10.1016/0092-8674(94)90280-1

[38] Smits C., Chechik M., Kovalevskiy O.V., Shevtsov M.B., Foster A.W., Alonso J.C., Antson A.A. Structural basis for the nuclease activity of a bacteriophage large terminase. EMBO Rep. 2009; 10:592–598. 19444313 10.1038/embor.2009.53 PMC2685612

[39] Hilbert B.J., Hayes J.A., Stone N.P., Xu R.-G., Kelch B.A. The large terminase DNA packaging motor grips DNA with its ATPase domain for cleavage by the flexible nuclease domain. Nucleic Acids Res. 2017; 45:3591–3605. 28082398 10.1093/nar/gkw1356 PMC5389665

[40] Roy A., Cingolani G. Structure of p22 headful packaging nuclease. J. Biol. Chem. 2012; 287:28196–28205. 22715098 10.1074/jbc.M112.349894 PMC3431676

[41] Thomsen N.D., Berger J.M. Running in reverse: the structural basis for translocation polarity in hexameric helicases. Cell. 2009; 139:523–534. 19879839 10.1016/j.cell.2009.08.043 PMC2772833

[42] Enemark E.J., Joshua-Tor L. Mechanism of DNA translocation in a replicative hexameric helicase. Nature. 2006; 442:270–275. 16855583 10.1038/nature04943

[43] Iyer L.M., Leipe D.D., Koonin E.V., Aravind L. Evolutionary history and higher order classification of AAA+ ATPases. J. Struct. Biol. 2004; 146:11–31. 15037234 10.1016/j.jsb.2003.10.010

[44] Ogura T., Whiteheart S.W., Wilkinson A.J. Conserved arginine residues implicated in ATP hydrolysis, nucleotide-sensing, and inter-subunit interactions in AAA and AAA+ ATPases. J. Struct. Biol. 2004; 146:106–112. 15095758 10.1016/j.jsb.2003.11.008

[45] Moreau M.J., McGeoch A.T., Lowe A.R., Itzhaki L.S., Bell S.D. ATPase site architecture and helicase mechanism of an archaeal MCM. Mol. Cell. 2007; 28:304–314. 17964268 10.1016/j.molcel.2007.08.013

[46] Kazmirski S.L., Podobnik M., Weitze T.F., O’Donnell M., Kuriyan J. Structural analysis of the inactive state of the Escherichia coli DNA polymerase clamp-loader complex. Proc. Natl. Acad. Sci. U.S.A. 2004; 101:16750–16755. 15556993 10.1073/pnas.0407904101 PMC529418

[47] Zhao Z., De-Donatis G.M., Schwartz C., Fang H., Li J., Guo P. An arginine finger regulates the sequential action of asymmetrical hexameric ATPase in the double-stranded DNA translocation motor. Mol. Cell. Biol. 2016; 36:2514–2523. 27457616 10.1128/MCB.00142-16 PMC5021374

[48] Mitchell M.S., Matsuzaki S., Imai S., Rao V.B. Sequence analysis of bacteriophage T4 DNA packaging/terminase genes 16 and 17 reveals a common ATPase center in the large subunit of viral terminases. Nucleic Acids Res. 2002; 30:4009–4021. 12235385 10.1093/nar/gkf524 PMC137109

[49] Mitchell M.S., Rao V.B. Novel and deviant Walker A ATP-binding motifs in bacteriophage large terminase-DNA packaging proteins. Virology. 2004; 321:217–221. 15051382 10.1016/j.virol.2003.11.006

[50] Bason J.V., Montgomery M.G., Leslie A.G., Walker J.E. How release of phosphate from mammalian F1-ATPase generates a rotary substep. Proc. Natl. Acad. Sci. U.S.A. 2015; 112:6009–6014. 25918412 10.1073/pnas.1506465112 PMC4434703

[51] Paschall C.O., Thompson J.A., Marzahn M.R., Chiraniya A., Hayner J.N., O’Donnell M., Robbins A.H., McKenna R., Bloom L.B. The Escherichia coli clamp loader can actively pry open the β-sliding clamp. J. Biol. Chem. 2011; 286:42704–42714. 21971175 10.1074/jbc.M111.268169 PMC3234947

[52] delToro D., Ortiz D., Ordyan M., Sippy J., Oh C.-S., Keller N., Feiss M., Catalano C.E., Smith D.E. Walker-A motif acts to coordinate ATP hydrolysis with motor output in Viral DNA packaging. J. Mol. Biol. 2016; 428:2709–2729. 27139643 10.1016/j.jmb.2016.04.029 PMC4905814

[53] Philo J.S. Is any measurement method optimal for all aggregate sizes and types?. AAPS J. 2006; 8:E564–E571. 17025274 10.1208/aapsj080365 PMC2761063

[54] Chistol G., Liu S., Hetherington C.L., Moffitt J.R., Grimes S., Jardine P.J., Bustamante C. High degree of coordination and division of labor among subunits in a homomeric ring ATPase. Cell. 2012; 151:1017–1028. 23178121 10.1016/j.cell.2012.10.031 PMC3652982

[55] Chemla Y.R., Aathavan K., Michaelis J., Grimes S., Jardine P.J., Anderson D.L., Bustamante C. Mechanism of force generation of a viral DNA packaging motor. Cell. 2005; 122:683–692. 16143101 10.1016/j.cell.2005.06.024

[56] Lee J.Y., Yang W. UvrD helicase unwinds DNA one base pair at a time by a two-part power stroke. Cell. 2006; 127:1349–1360. 17190599 10.1016/j.cell.2006.10.049 PMC1866287

[57] Holm L., Sander C. Dali: a network tool for protein structure comparison. Trends Biochem. Sci. 1995; 20:478–480. 8578593 10.1016/s0968-0004(00)89105-7

[58] Dixit A.B., Ray K., Thomas J.A., Black L.W. The C-terminal domain of the bacteriophage T4 terminase docks on the prohead portal clip region during DNA packaging. Virology. 2013; 446:293–302. 24074593 10.1016/j.virol.2013.07.011 PMC3903156

[59] Lin H., Rao V.B., Black L.W. Analysis of capsid portal protein and terminase functional domains: interaction sites required for DNA packaging in bacteriophage T4. J. Mol. Biol. 1999; 289:249–260. 10366503 10.1006/jmbi.1999.2781

