# Supplementary material for: Structure of the large terminase from a hyperthermophilic virus reveals a unique mechanism for oligomerization and ATP hydrolysis
Source: Nucleic Acids Res. 2017 Oct 24;45(22):13029–42. doi: 10.1093/nar/gkx947 (PMC5727402; doi:10.1093/nar/gkx947)
Supplement: Supplementary Data [file gkx947_supp.pdf]

Supplementary information for:

Structure of the large terminase from a hyperthermophilic virus reveals a unique mechanism for oligomerisation and ATP hydrolysis

Rui-Gang Xu, Huw T. Jenkins, Alfred A. Antson, Sandra J. Greive

Supplementary Figures

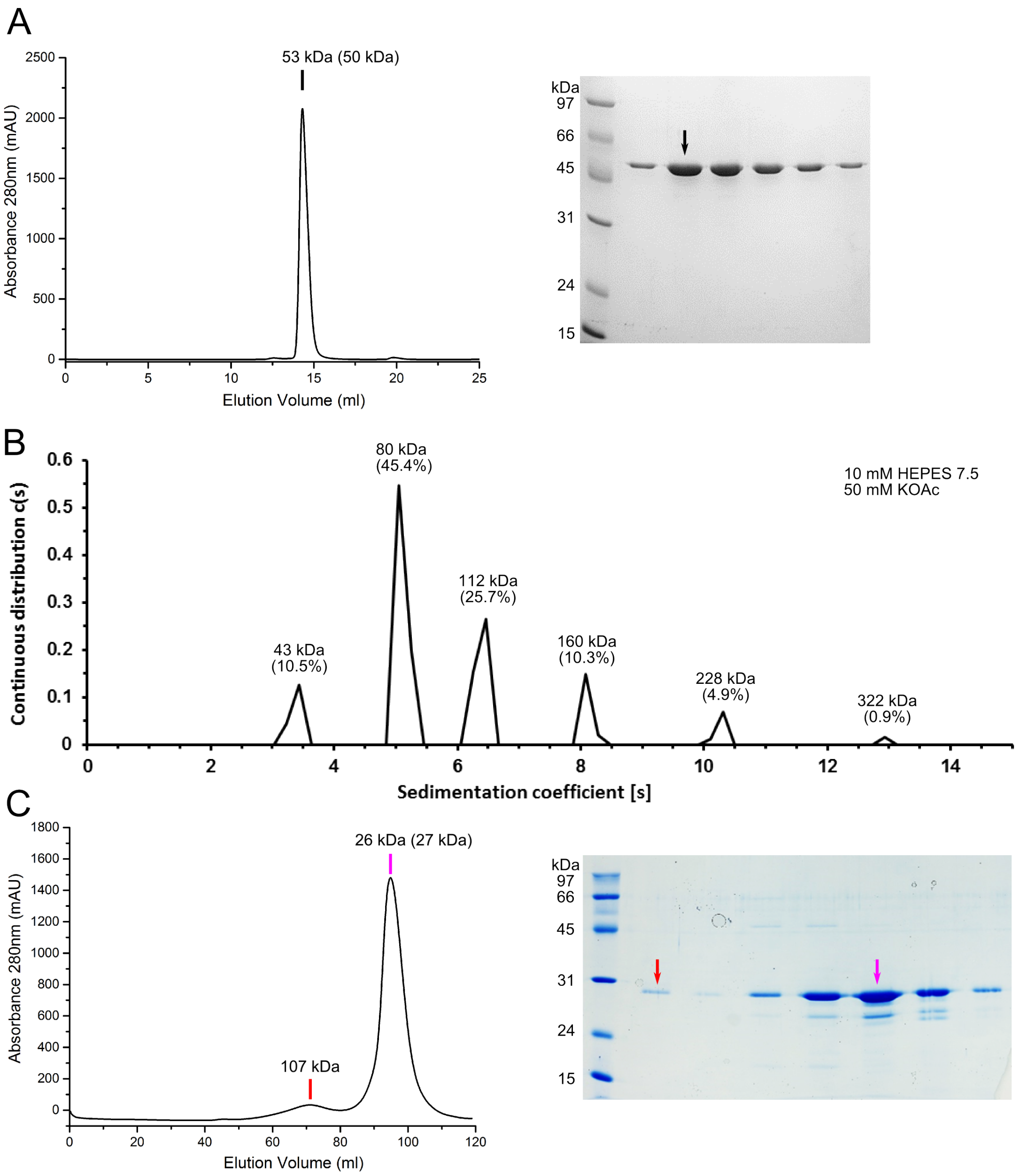

Supplementary Figure 1. Oligomer state. (A) Estimation of the molecular weight of large terminase (1-427) by analytical size exclusion chromatography (left) and SDS-PAGE (right). (B) Oligomer states of the full-length large terminase in sedimentation

velocity analytical ultracentrifugation (SV-AUC). The molecular weight and ratio of the monomer and different oligomer states were indicated. The frictional ratio of the observed oligomers is fitted as 1.28. In SV-AUC, analysis of associating systems with rapid kinetics, the peaks representing the different oligomeric species are often broader and shifted towards the monomer species ~45 kDa. As a result, the calculated molecular weight for higher order oligomers may tend to be underestimated compared to the theoretical value, as observed for 4 prominent oligomer peaks (Brown et. al. (2008) Curr Protoc Immunol. Chapter 18: Unit 18.15). This would suggest that the small species at ~13 S reflects a non-specific aggregation (>7 monomers), consistent with the observation that the protein exhibits concentration dependent aggregation in low salt buffers. (C) Estimation of the molecular weight of the N-terminal ATPase domain (1-234). The corresponding fractions of the oligomer and monomer peaks (indicated by coloured arrows) observed by size exclusion chromatography (left) were analysed by SDS-PAGE (right).

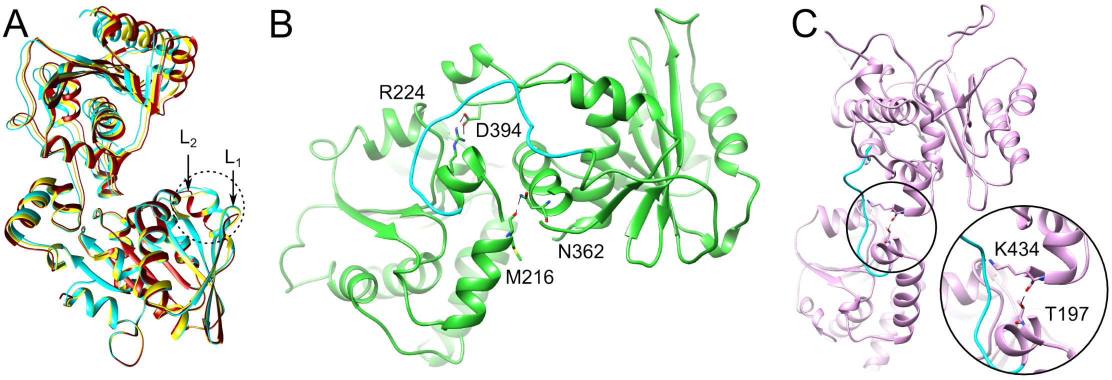

Supplementary Figure 2. Structure comparison and interdomain hydrogen bonds. (A) Superposition of the D6E large terminase ATPase domains from the different molecules within an asymmetric unit. An α-helix which adopts different conformations is highlighted using a dashed circle. The positions of loops L_1 and L_2 are indicated. The electrostatic interactions between the ATPase and the nuclease domain are shown in sticks for (B) D6E and (C) Sf6 large terminase. The linker which tethers the two domains together are coloured in cyan.

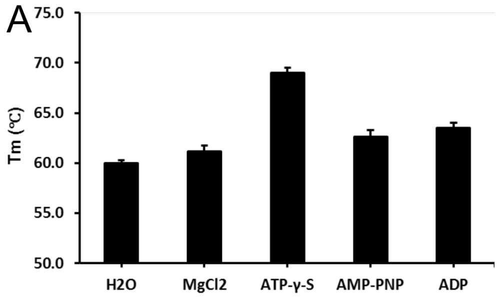

Mg^{2+}	-	+	+	+	+
Additive	-	-	ATP-γ-S	AMPPNP	ADP
Tm (°C)	60.0	61.1	69.0	62.6	63.4
ΔTm (°C)	-1.1	-	7.8	1.4	2.3

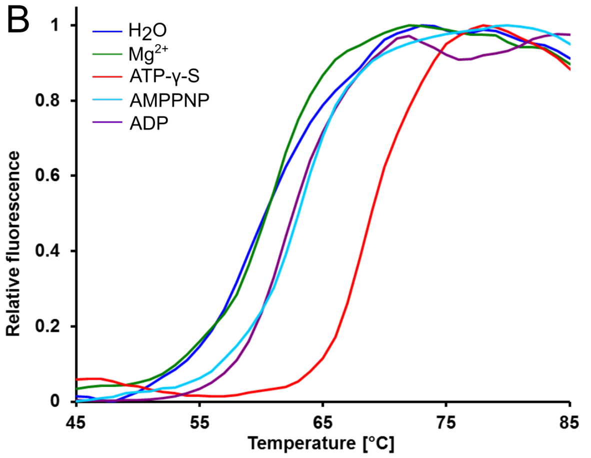

Supplementary Figure 3. Protein stabilisation by binding of ATP analogues. (A) Plot of the observed melting temperatures in the presence and absence of ADP, ATP and nonhydrolyzable ATP analogues, showing the average and standard deviation for three independent experiments (B) Representative melting curves are shown within a temperature range of 45-85˚C.

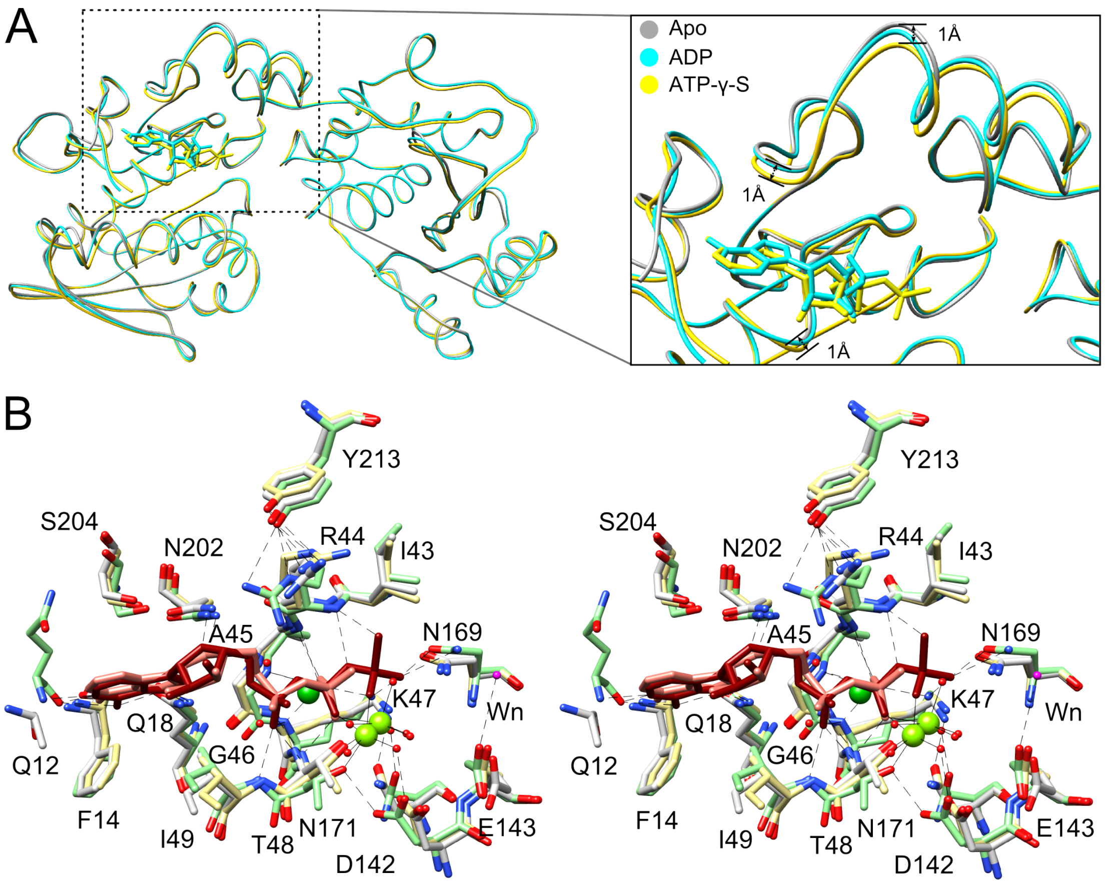

Supplementary Figure 4. ATPase active site and conformational changes. (A) Superposition of the apo (grey), ADP (cyan) and ATP-γ-S (yellow) complex structures. ADP and ATP-γ-S are shown as sticks. Cα rmsd differences are indicated after superposition of the central β-sheet of the ATPase domain. (B) Stereo diagram showing superposition of all three structures from above. Hydrogen bonding interactions on all diagrams are in black dashed lines, coordination to the metal (Mg^{2+}, green sphere) is shown by solid black lines.

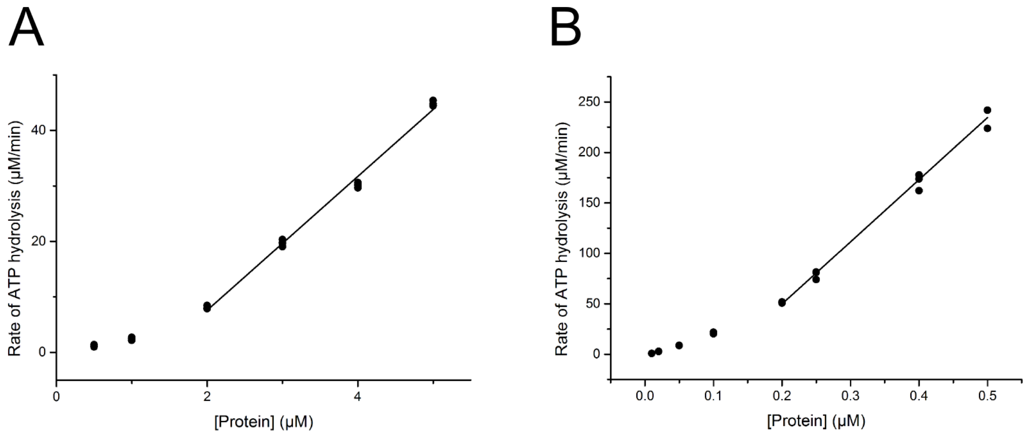

Supplementary Figure 5. ATP hydrolysis. Rate of ATP hydrolysis of (A) full-length wild type D6E large terminase protein and (B) ATPase domain was determined by coupled ATPase assays at various protein concentrations (Full-length: 0.5-5 μM; ATPase: 0.01-0.5 μM).

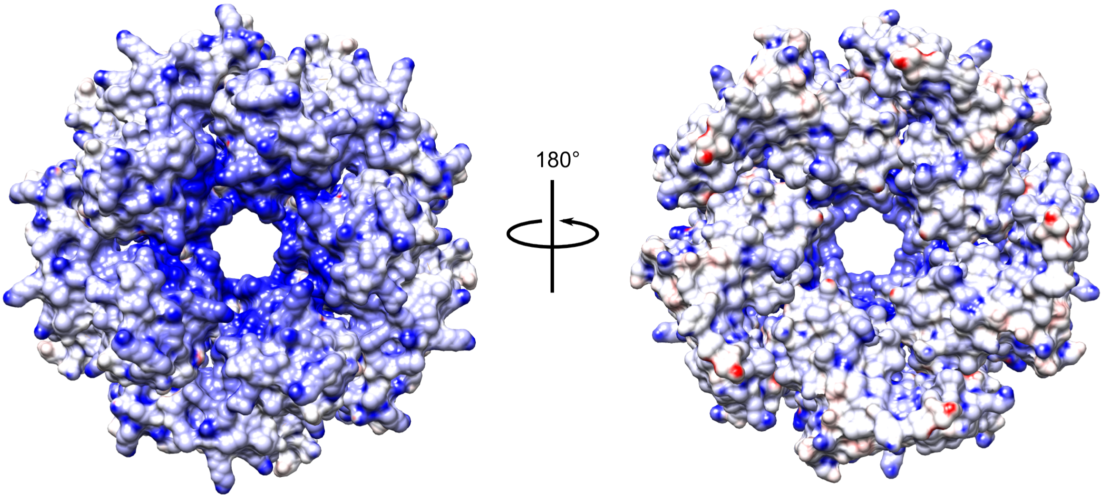

Supplementary Figure 6. Surface electrostatic potentials. Continuum electrostatics calculations of the D6E large terminase pentameric ATPase model. Surface electrostatic charge potential is shown at pH 7.0. Positive charge is shown in blue while negative charge is shown in red.

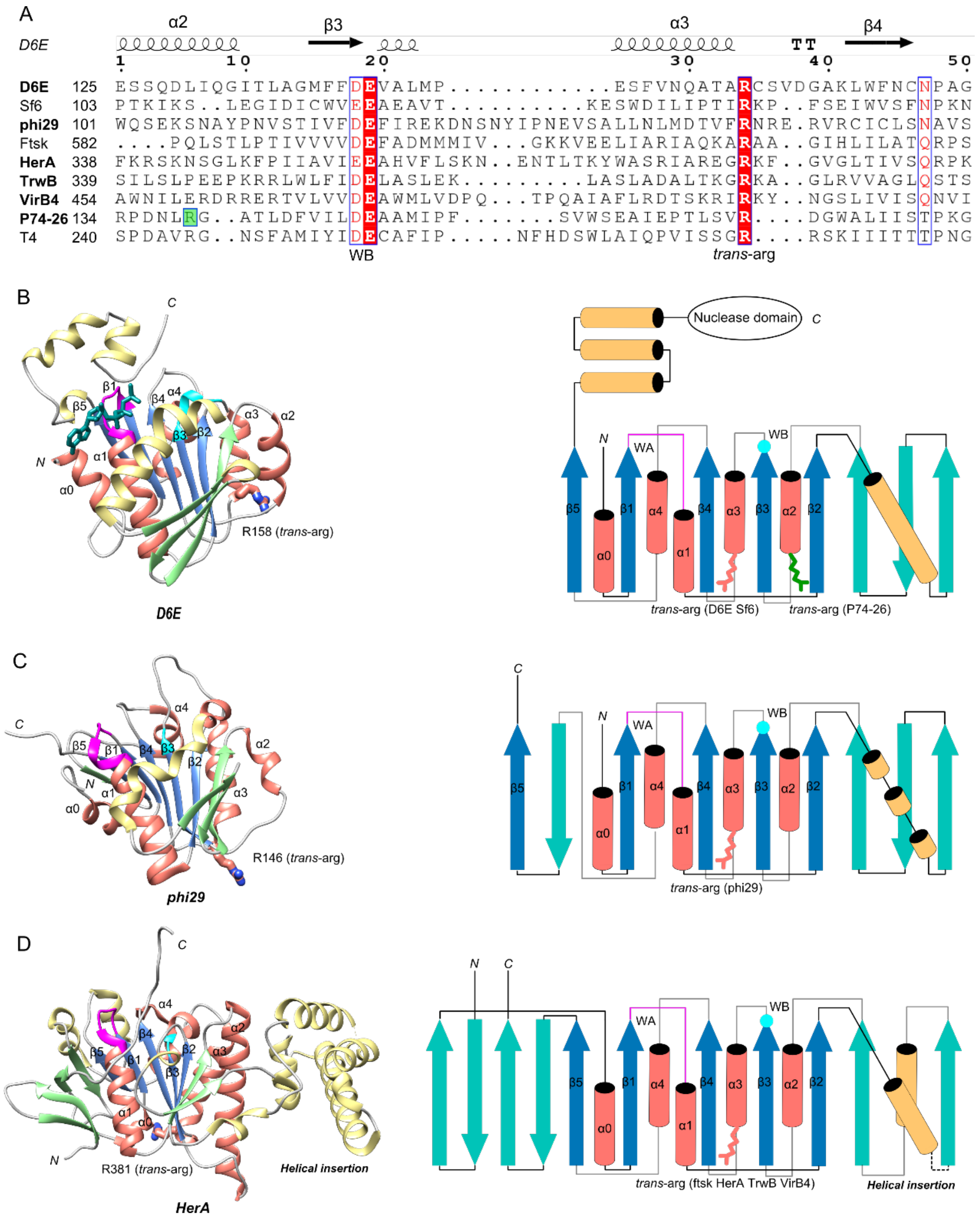

Supplementary Figure 7. Comparison of *trans*-arginine fingers. (A) Structure based sequence alignment of the D6E large terminase ATPase with phi29 packaging ATPase, large terminase ATPases from bacteriophage Sf6, T4, P74-26 and Ftsk/HerA superfamily ATPases including Ftsk, HerA, TrwB and VirB4. Proteins for which the *trans*-acting arginine finger residues have been characterised biochemically are highlighted in bold (P74-26 residue highlighted in green). The structure and topological diagram for D6E large terminase ATPase, phi29 ATPase and HerA are shown in (B) (C) and (D). Secondary structures are coloured in salmon (α-helix), blue (β-strand) and grey (coil) for the canonical ASCE fold, while yellow (α-helix) and green (β-strand) are used for the insertions. ATP is coloured in dark cyan (B). Walker A (megata) and Walker B (cyan) represent the Walker A and B motifs, respectively. The position of the *trans*-acting arginine is indicated.

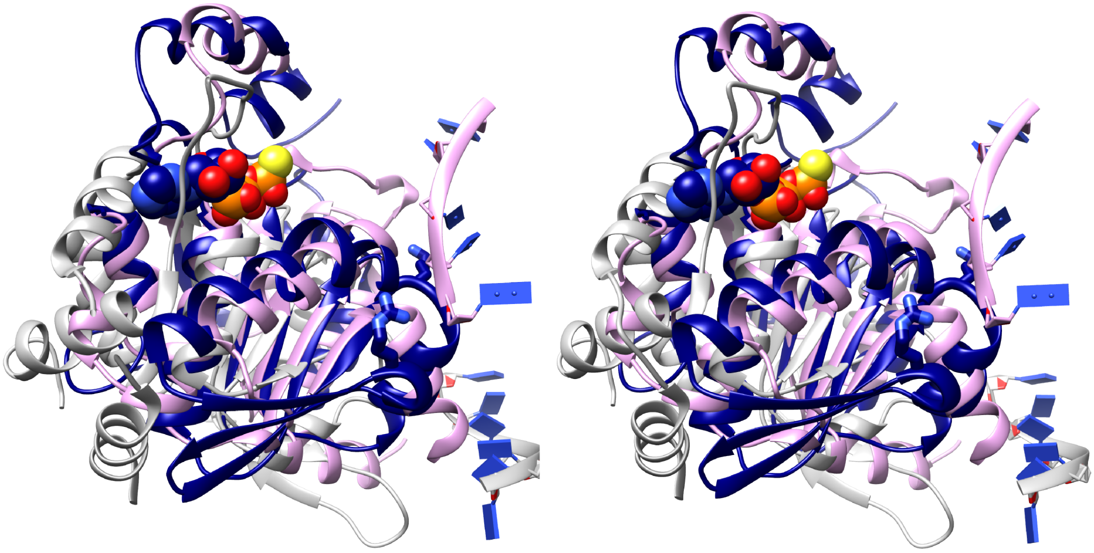

Supplementary Figure 8. Structural superposition. Stereo view of the superposition of D6E large terminase ATPase (navy blue) with E1 helicase (grey) and UrvD helicase (light pink) with bound DNA. Loop L_1 and L_2 (thick ribbons) of the D6E large terminase ATPase is in proximity to the DNA (square bases). R101 and K123 are shown in thick sticks. The bound ATP-γ-S is shown in spheres.

Supplementary Table 1. X-ray data collection and refinement statistics.

**Crystal form**	**1**<br>**Peak**	**1**<br>**Inflection**	**1**<br>**High energy remote**
Wavelength (Å)	1.8926	1.8942	1.7101
Space group	R32	R32	R32
Unit-cell a, b, c (Å)	103.3, 103.3, 268.9	103.6, 103.6, 269.3	103.8, 103.8, 268.8
Unit-cell α, β, γ (˚)	90.0, 90.0, 120.0	90.0, 90.0, 120.0	90.0, 90.0, 120.0
Resolution(Å)	46.09-2.90 (3.08-2.90)	46.18-2.90 (3.08-2.90)	46.13-2.90 (3.08-2.90)
Rmerge (%)	9.7 (114.3)	8.2(123.4)	7.2(109.5)
I/σ<I>	12.3(1.8)	13.8(1.7)	15.9(2.0)
Completeness (%)	100(99.9)	99.7(98.4)	99.8(99.1)
Multiplicity	9.4(9.0)	9.4(8.8)	9.0(8.5)
$CC_{1/2}$	0.996(0.695)	0.998(0.660)	0.999(0.664)

Values in the parentheses are for the outermost resolution shell.
MAD, multi-wavelength anomalous diffraction.

Supplementary Table 2. Comparison of intra-domain electrostatic interactions.

Electrostatic interactions / Large terminase	ASCE subdomain-nuclease domain	Lid subdomain-nuclease domain
D6E	M216-N362 (1)	R224-D394 (2)
T4	-	-
Sf6	-	K434-T197 (1)

Supplementary Table 3. Comparison of intra-domain VDW interaction areas.

Areas of VDW interaction / Large terminase	ASCE subdomain-nuclease domain	Lid subdomain-nuclease domain	Total between ATPase and Nuclease domains
D6E	119	243	362
T4	196	6	212
Sf6	30	48	78
